# Peptide Conjugates with Small Molecules Designed to Enhance Efficacy and Safety

**DOI:** 10.3390/molecules24101855

**Published:** 2019-05-14

**Authors:** Rongjun He, Brian Finan, John P. Mayer, Richard D. DiMarchi

**Affiliations:** 1 Novo Nordisk Research Center, Indianapolis, IN 46241, USA; bfin@novonordisk.com; 2 Department of Molecular, Developmental & Cell Biology, University of Colorado, Boulder, CO 80309, USA; John.Mayer@Colorado.edu; 3 Department of Chemistry, Indiana University, Bloomington, IN 47405, USA

**Keywords:** peptide, peptide-drug conjugate, mixed-mode pharmacology, GLP-1, GnRH, LHRH, chemical linker, cancer, diabetes, obesity, drug discovery

## Abstract

Peptides constitute molecular diversity with unique molecular mechanisms of action that are proven indispensable in the management of many human diseases, but of only a mere fraction relative to more traditional small molecule-based medicines. The integration of these two therapeutic modalities offers the potential to enhance and broaden pharmacology while minimizing dose-dependent toxicology. This review summarizes numerous advances in drug design, synthesis and development that provide direction for next-generation research endeavors in this field. Medicinal studies in this area have largely focused upon the application of peptides to selectively enhance small molecule cytotoxicity to more effectively treat multiple oncologic diseases. To a lesser and steadily emerging extent peptides are being therapeutically employed to complement and diversify the pharmacology of small molecule drugs in diseases other than just cancer. No matter the disease, the purpose of the molecular integration remains constant and it is to achieve superior therapeutic outcomes with diminished adverse effects. We review linker technology and conjugation chemistries that have enabled integrated and targeted pharmacology with controlled release. Finally, we offer our perspective on opportunities and obstacles in the field.

## 1. Introduction

Peptides represent a powerful class of medicine that currently serves multiple diseases and often constitutes indispensable, life-preserving pharmacology [1,2,3,4]. They often display exquisite affinity and specificity for a unique molecular target. This coupled with straightforward endogenous metabolism to constituent amino acids typically translates to high potency medicines, with minimal off-target adverse effects. Being of modest molecular size and certainly much smaller than most proteins enables the relationship of peptide structure to function to be rapidly interrogated by synthetic methods that have matured over the last fifty years [5,6,7,8,9,10]. These synthetic methods have also evolved to achieve success at a commercial scale which is a significant advantage as it enables molecular diversity that is not readily achieved in larger molecules, facilitates translation to clinical studies and yet often nicely integrates with biosynthetic approaches for larger production and reduced cost. Prominent examples include insulin and related analogs, glucagon-like peptide 1 agonists (GLP-1), somatostatins and many others [1,2,3,4,11,12,13,14]. Accordingly, peptide-based drug candidates much like proteins have recorded a higher success rate in commercial development relative to classical small molecules. Novo-Nordisk and Amgen, which have heavily focused on peptide and protein drugs, reported the highest clinical success rates relative to similarly-sized peer companies in 2016 [15]. Multiple factors, however, influence these results, such as disease selection, portfolio decision making and executive appetite for risk [16,17].

More than sixty peptide-based drugs are commercially marketed globally, with more than a hundred in various stages of commercial development and many, many more in preclinical research. Virtually all disease areas are touched at some level with endocrinology, cancer, infectious and cardiovascular diseases being most prevalent [1,2,3,4]. The global sales for peptide-based medicines in 2015 were in excess of fifty billion U.S. dollars and forecasted to reach seventy in 2019 [1]. Among these, insulin-related medicines are by far the largest given the global epidemic of maturity-onset diabetes [18]. There are many billion-dollar drugs and notably, the use of multiple GLP-1 agonists is accelerating rapidly [19]. It is clear that peptides fulfill a unique therapeutic need where traditional small molecules have not.

Similar to drug discovery directed at small molecules, peptide research has evolved in the direction of multimode pharmacology, [20,21,22] where single molecules activate multiple receptors in an additive and occasionally in a synergistic manner to achieve superior efficacy often at reduced dose [1,2,3,4,23,24,25]. This type of pharmacology is exemplified in purposefully integrated, dual agonism at amylin and calcitonin, GLP-1 and glucagon, or with gastric inhibitory peptide (GIP), and triple agonism at GLP-1, glucagon and GIP in treatment of the metabolic syndrome [26,27,28,29,30,31,32,33,34,35]. The sequence of these multi-action peptides largely derives from intermixing resides from each native hormone to achieve balanced, full agonism at the respective cognate receptors. It is the inherent structural similarity within these related receptors and their natural ligands that enables the discovery of chimeric peptides that can promiscuously bind more than once receptor with similar affinity. Consequently, there are limits to where this approach can be successfully applied as hormones of a more distant sequence will prove increasingly difficult, if not impossible to successfully integrate to a single common binding face. In those instances where the respective receptors are too distant to assemble a single ligand that can fulfill high-affinity binding more traditional approaches to functionate through chemical conjugation to heterodimeric and higher polymeric forms have been applied. This approach is commonly employed in antibody-based drug candidates where more than one receptor is blocked [36,37,38]. Although less elegant in their molecular design and resulting in appreciably increased molecular size, such polypeptide conjugates can similarly bestow the pharmacological benefits of peptides with a single hybridized binding site.

Conjugates of peptides and small molecules empower the virtues of peptide-based pharmacology with traditional medicinal chemistry [1,2,3,39,40,41,42,43,44,45,46]. The result is a macromolecule, and as such the biophysical character of the drug candidate and the resultant properties for patient use have paralleled what has been advanced in peptide and protein therapeutics. Consequently, the progression of this form of medicinal chemistry has evolved more from the large molecule side to embrace small molecules, than vice versa. In this review, we focus on peptide-drug conjugates that promote the integrated benefits of peptides and smaller, non-peptide pharmacophores. The presentation is intended to supplement reviews focusing exclusively on peptide-based therapeutics [1,2,3] and complement those that specifically emphasize applications in cancer [40,41]. The reader is also directed to other reviews with an emphasis on physiochemical properties of peptide-drug conjugates [42] and those predominantly employed for optimizing pharmacokinetic performance [43]. Finally, protein-based drug conjugates other than antibodies are not reviewed but can be found elsewhere [44], and similarly so conjugates for diagnostic purposes with imagining agents or organometallic entities [45,46]. We have selectively cited prominent examples of peptide-drug conjugates as representatives of the class to offer our perspective in molecular design, selection of covalent linkers, and other aspects that influence performance.

## 2. Why Peptide-Drug Conjugates?

Peptide-based therapeutics historically represented a small fraction of conventional pharmaceutical discovery research where the emphasis has been on small molecules that prioritized the convenience in oral administration nearly as much as the efficacy of the drug. This has resulted in an excessive investment on a finite number of high-profile drug targets that have constrained the broader exploration of human pathology [47,48]. Peptides as a molecular class are well recognized to often provide unprecedented efficacy and the attempts to reduce them to structural mimetics that could be orally administered have largely failed, despite sizable investment to do so. The advent of rDNA-based protein drugs, and in particular antibodies have demonstrated the importance of drug efficacy, especially when applied to life-altering diseases to dominate the convenience of oral administration. Tangential to the popularity of protein-based therapeutics has been increased attention for peptide therapeutics. As a result, notable successes such as parathyroid hormone (PTH), GLP-1, GLP-2 agonists complement the historical importance of such peptides as insulin, gonadotropin-releasing hormone, somatostatin, calcitonin and numerous other less prominent entities such as glucagon, vasopressin and oxytocin. Nonetheless, there is a general sense within the peptide community of there being too few validated drug targets. The integration of peptides with traditional small molecules provides a venue to advance novel macromolecular therapeutics that provide supplemental efficacy but also addresses intracellular drug targets as it has constituted a central limitation in peptide and protein-based pharmacology.

Small molecule drug candidates have historically recorded a higher attrition rate in clinical development, which partially results from suboptimal physicochemical properties [16,17,49]. Conjugation to peptides is an approach to address poor aqueous solubility, untimely metabolism and potentially facilitate cell permeability. It has provided targeted delivery of small molecules to diseased tissue to enhance local drug concentration, and mitigate toxic effects arising from systemic exposure and accumulation in non-diseased tissues [1,2,3,4,50,51]. Drug-Drug Interactions (DDI) constitute a common cause of adverse drug reactions that undermines efficacy and with the increased use of multiple drugs in the treatment of complex diseases has emerged as something of elevated importance in drug development [52]. Peptide-drug conjugates by design can minimize DDI by lessening accumulation in tissues where inappropriate biological action is adversely increasing pharmacology arising from other drugs. In this regard, peptide-drug conjugates are largely confined to the extracellular space and as such have been reported to minimize inappropriate hepatic metabolism [2,51].

The origin in the design of molecular conjugates can be traced as far back as a century ago when the German physician-scientist Paul Ehrlich coined the term ‘magic bullet’ in characterizing a cytotoxic drug to be selectively delivered to a tumor via a targeting agent [53,54]. It was in the second half of the last century when several examples were reported [55,56,57,58], with the first instance employing methotrexate (MTX) conjugation to an antibody directed against leukemia cells [55]. The first clinical trial of such an antibody-directed cytotoxic agent (ADC) was reported in 1983 [59], in which an anti-carcinoembryonic antigen (CEA) antibody directed a vinca-alkaloid in treatment of advanced stage cancer. Nearly two decades later as we entered this century the first ADC was FDA-approved and named gemtuzumab ozogamicin [60]. With a commercial trade name of Mylotarg, this chemical conjugate consists of an anti-CD33 antibody linked with calicheamicin, a drug of high systemic toxicity for the treatment of acute myeloid leukemia (AML). The extended period from Paul Ehrlich’s time to first drug registration of an ADC was to an appreciable degree due to the relative immaturity of antibody-based therapeutics, until the advent of the last decade of the twentieth century [61]. The first ADC employed polyclonal antibodies with cytotoxic agents non-covalently associated, with human sequence antibodies emerging, with maturation of rDNA-based expression [61]. Subsequent to Mylotarg, brentuximab vedotin (Adcetris) received regulatory approval in 2011 for treatment of Hodgkin lymphoma and systemic anaplastic large cell lymphoma [62,63], and trastuzumab emtansine (Kadcyla) was similarly approved in 2013 for the treatment of HER2-positive metastatic breast cancer [61,64]. Mylotarg was subsequently withdrawn from distribution in 2010 for safety concerns and the absence of proven clinical benefit in a follow-up clinical trial [65]. It was successfully reintroduced as Besponsa in 2017 for treatment of AML and in addition treatment of relapsed or refractory acute lymphoblastic leukemia (ALL) [66]. Currently, there are more than one hundred registered clinical studies that employ some form of ADC [67].

Despite the initial clinical successes, multiple challenges exist in the development of next-generation ADCs. Issues pertaining to the continued identification and validation of disease-specific target antigens remain a central biological challenge to the approach. Advances in site-specific chemical conjugation, often employing novel orthogonal conjugation chemistries with linkers designed for improved therapeutic index continue to provide more homogenous drug candidates. The large molecular size of antibodies has raised questions pertaining to efficient distribution and delivery to disease tissues with subsequent cellular transport. In these last domains, peptide conjugates given their inherently smaller size possess an inherent advantage in comparison to antibodies. The increased molecular diversity and accuracy in chemical synthesis of peptides provide structural precision and optimization that exceeds what is possible with antibodies [60,61,62,63,68,69]. As such medicinal chemistry has been employed to enhance potency, drug distribution, pharmacokinetics and metabolism. This approach also simplifies commercial synthesis and compliance with regulatory demands for registration and subsequent requirements in drug manufacture [70]. Peptide-based drug conjugates bind to cell surface targets with high-affinity that parallels that of antibodies and recognize a broad spectrum of biological targets, most notably G protein-coupled receptors (GPCRs), receptor tyrosine kinases (RTKs), and integrins. Being of significantly reduced molecular size relative to ADCs, there is potential for more efficient delivery to sequestered-targets leading to enhanced efficacy, and reduced immunogenicity [1,2]. It should be noted that peptide-drug conjugates are pharmacokinetically distinct from antibodies with a much-reduced circulation time. This can be of particular advantage in the delivery of highly toxic reagents where extended exposure is unwarranted. However, it initially proved a disadvantage as treatment of solid tumors more often required sustained pharmacokinetics, of the type inherent to antibodies. The maturation in chemical approaches to alter and even tailor time-action of peptides with chemical lipidation, pegylation and a host of other technologies has largely eliminated this difference relative to antibodies. While the comparisons will continue, it is less a question of which molecular platform is superior than celebrating diversity as a tool to be employed in achieving superior disease outcomes, conveniently administered at a suitable financial cost.

## 3. Approved Peptide-Drug Conjugates

Somatostatin (growth hormone-inhibiting hormone, GHIH) is a peptide produced by paracrine cells in the gastrointestinal tract, pancreatic delta cells, and hypothalamic neurons to control multiple endocrine functions [71,72]. It inhibits secretion of growth hormone, thyroid stimulating hormone, and other pituitary-derived hormones, as well as hormone secretion from pancreatic and gastrointestinal cells. Somatostatin exhibits many direct and indirect effects to suppress growth and differentiation in several different cancer cells. Somatostatin analogs such as Octreotide, Lanreotide, and Pasireotide are clinically used in the treatment of acromegaly, as well as hormone-dependent tumors such as pancreatic, and vasoactive intestinal peptide-secreting tumors [72,73]. Somatostatin analogs have also been clinically studied in breast, lung, prostate and gastrointestinal cancers [73].

**Figure 1:**
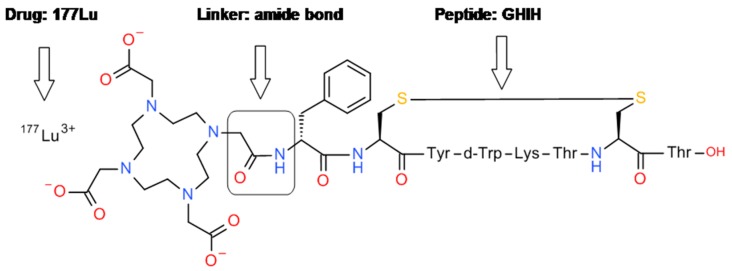
Chemical structure of growth hormone-inhibiting hormone (GHIH)-177Lu conjugate (177Lu-Dotatate).

Somatostatin biologically functions through a family of related receptors in the GPCR superfamily [71]. There are five somatostatin receptors subtypes (SSTR1-5) that are differentially expressed in brain cortex, pituitary, adrenals, pancreas, heart, and gastrointestinal tract [72,74]. The SSTR2 is reported to be overexpressed in many tumors and undergoes ligand-induced internalization. This latter property renders SSTR2 a potential target for intracellularly delivering cytotoxic and other growth suppressive agents to tumor cells [71]. Various somatostatin analogs have been conjugated to radioactive chemotherapeutic agents to induce tumor death by a process termed peptide receptor radionuclide therapy (PRRT) [75,76]. Targeted radioisotope therapy complements the inherent anticancer pharmacology of somatostatin, while simultaneously reducing the systemic radioactive toxicity. Radioactive isotopes conjugated to somatostatin include beta-emitter nuclide 90Y, gamma-emitter 111In, beta and gamma emitter 177Lu, and other more commonly employed nuclear medicines [75,76]. PRRT inhibits tumor progression [77,78,79], and recently a 177Lu Dotatate conjugate was approved as for treatment of gastroenteropancreatic neuroendocrine tumors [80,81]. 177Lu as a beta-emitter exhibits maximal tissue penetration less than 2 mm, which renders it a good irradiation choice for small tumors. The Lutetium isotope has a reasonable physical half-life of 6.7 days, which makes it favorable from a therapeutic and safety perspective as 99% of the drug is eliminated within two weeks [82,83,84]. The 177Lu is chelated to octreotide, a somatostatin analog through a DOTA high-affinity binder that is covalently linked to the hormone (Figure 1) [76,85]. The peptide exhibits high potency (IC_50_: 1.5 nM) at SSTR2 with greater than a hundredfold selectivity over SSTR5 (IC_50_: 547 nM) and SSTR3 (IC_50_: >1000 nM) [86]. In a single-arm clinical trial in 310 patients with gastroenteropancreatic cancer, 177Lu-Dotatate treatment provided partial tumor remissions in 28% of patients and complete remissions in 2%. The median progression-free survival was 33 months [87]. In a recent phase 3 trial in progressing, advanced midgut neuroendocrine tumors 177Lu-Dotatate treatment resulted in a progression-free survival rate of 65.2% versus 10.8% at twenty months relative to continuing somatostatin treatment alone [88]. The 177Lu-Dotatate exhibited limited hemato-toxicity, but without renal toxicity. These results in advanced refractory cancer demonstrate the much-improved therapeutic efficacy and safety of the peptide-drug conjugate in comparison to somatostatin or radioisotope therapy alone.

## 4. Representative Peptide-Drug Conjugates in Clinical Development

### 4.1. GnRH-Doxorubicin Conjugate

Gonadotropin-releasing hormone (GnRH or LHRH) is a hypothalamic peptide hormone that binds to receptors in the anterior pituitary to stimulate the release of the follicle-stimulation hormone and luteinizing hormone, two hormones seminal to reproduction [89,90]. GnRH also stimulates gonadotropin release and subsequent steroid hormone release, which are purported stimulants to many forms of cancer [91,92]. Continuous GnRH receptor activation causes down-regulation and desensitization to reduce endogenous steroid hormone biosynthesis and release [93]. Consequently, GnRH super-agonists have been successfully employed in hormone-dependent cancers in what is termed androgen deprivation therapy (ADT) [92,94].

**Figure 2:**
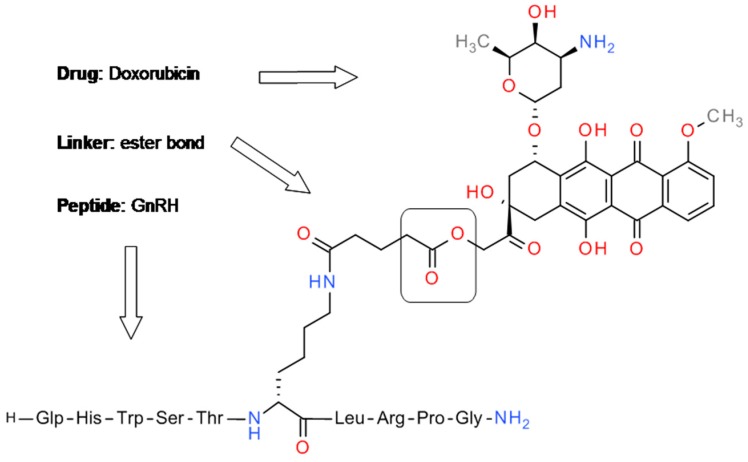
Chemical structure of gonadotropin-releasing hormone (GnRH) or luteinizing hormone-releasing hormone (LHRH)-doxorubicin conjugate (Zoptarelin Doxorubicin, AN-152, AEZS-108).

The GnRH receptor is expressed in many endocrine cancers, including breast, ovarian, endometrial, and prostate tumors. Its presence provides the means to target oncolytic drugs to these cancer cells to supplement the clinical benefits currently achieved with ADT alone [90,95]. Zoptarelin Doxorubicin (AN-152, AEZS-108, Zoptrex^TM^) is a peptide-drug conjugate composed of a GnRH analog and doxorubicin through an ester bond with a glutaric acid spacer (Figure 2) [96]. The conjugate proved more effective than doxorubicin in inhibiting cell proliferation in GnRH receptor positive cancer cell lines [96]. It also was more potent than either agent alone in several xenograft mouse tumor models [97]. These results validate the virtue of targeted, complementary GnRH and doxorubicin pharmacology. Phase 1 studies in endometrial, ovarian and prostate cancer established Zoptarelin Doxorubicin’s safety, pharmacokinetics, and maximum tolerated dose [98,99,100]. In several phase 2 studies the drug-conjugate exhibited promising clinical activity with low systemic toxicity in castration and taxane-resistant prostate cancer [101], advanced or recurrent endometrial cancer [102], and platinum refractory ovarian cancer [103]. In a recent large phase 3 registration trial in advanced endometrial cancer Zoptarelin Doxorubicin disappointedly failed to improve median overall survival, or progression-free survival when compared to standard doxorubicin therapy [104]. The basis of the failure is unknown but given that doxorubicin at highest dose did not significantly improve patient survival there is a suspicion that deficiencies specific to doxorubicin might be the primary cause, as opposed to something inherent to the drug-conjugate.

### 4.2. Angiopep-2-Paxlitaxel Conjugate

**Figure 3:**
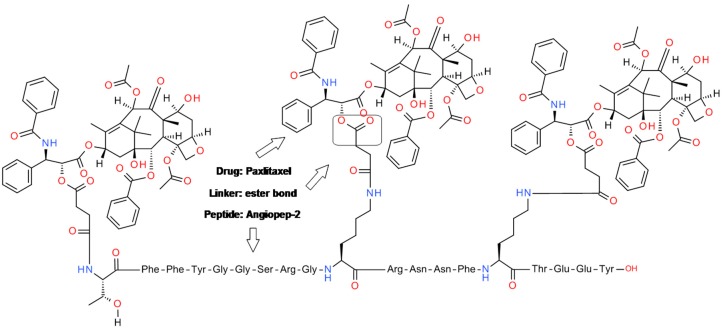
Chemical structure of angiopep-2-paxlitaxel conjugate (ANG1005, GRN1005).

Paclitaxel is a potent oncolytic drug that has been widely used in several different cancers [105,106]. However, its low blood–brain barrier (BBB) permeability coupled with multidrug resistance efflux by P-glycoprotein pump (P-gp) has resulted in limited activity in primary and metastatic brain tumors. Angiopep-2 is a peptide that binds the low-density lipoprotein receptor-related protein 1 (LPR1) and it is upregulated in many tumors, including glioma [107,108]. ANG1005 (also named GRN1005) (Figure 3) is a drug conjugate composed of angiopep-2 and paclitaxel designed to increase brain transport through LPR1 mediated transcytosis [109]. The ANG1005 conjugate includes as many as three molar equivalents of paclitaxel relative to a peptide with intent to maximally increase cytotoxic drug concentration [110,111]. ANG1005 demonstrated excellent cytotoxicity against glioblastoma, lung and ovarian cancer cells. Furthermore, ANG1005 was effectively transported to the brain in an LPR1-dependent manner [112] and appeared unaffected by P-gp efflux that would otherwise impair therapeutic efficacy. The efficacy was established through in vivo studies where ANG1005 significantly prolonged survival in mice bearing xenografted glioblastoma or lung carcinoma cells [109]. In phase 1 clinical trials in recurrent malignant glioma tumors [110,111], ANG1005 exhibited plasma half-life of 3.6 h and was well tolerated with a toxicity similar to paclitaxel. Importantly, ANG1005 is designed to cross the BBB to deliver therapeutic concentrations of paclitaxel to the tumor site. A phase 2 study in breast cancer patients with brain metastases demonstrated in a subpopulation of patients a favorable median survival time of eight months as compared to four with standard treatment achieved with other forms of therapy, or two months without treatment [113]. Several additional phase 2 studies with ANG1005 have completed, and include recurrent high-grade glioma, non-small cell lung and brain metastases [114]. Currently, ANG1005 has been successfully registered as an orphan drug for the treatment of multiform glioblastoma [115], and a phase 3 clinical trial with ANG1005 against leptomeningeal disease from breast cancer is reported to be in recruitment phase [116].

### 4.3. Tetrapeptide-Thapsigargin Conjugate

**Figure 4:**
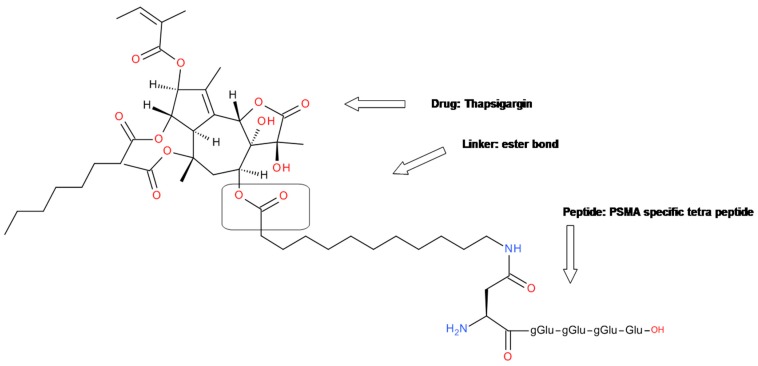
Chemical structure of tetrapeptide-thapsigargin conjugate G202 (Mipsagargin).

Thapsigargin is a highly potent cytotoxic natural product that induces apoptosis in mammalian cells by binding the sarco/endoplasmic reticulum calcium ATPase (SERCA) to disrupt the Ca^2+^ gradient across cytosolic and reticulum compartments [117]. Unlike other cytotoxic agents which inhibit rapidly proliferating cells, thapsigargin kills in a less specific manner given its mechanism, and this has undermined its potential as a chemotherapeutic agent. Chemical conjugation of thapsigargin to a tetrapeptide yields a charged conjugate termed G202 (Mipsagargin) (Figure 4) that is unable to cross the cell membrane to reach SERCA [118,119,120]. The tetrapeptide is a substrate of the membrane-bound proteolytic enzyme prostate-specific membrane antigen (PSMA), which is overexpressed in prostate cancer and other tumors, but much less so in normal tissues [121,122]. The tetrapeptide is processed by PSMA to provide an analog that is now cell permeable, cytotoxic, and extracellularly concentrated adjacent to cancerous prostate cells [118,119,120]. By in vitro assessment, G202 was reported to be 57-fold more potent in cell proliferation assays in human prostate cancer cells expressing PMSA, implying PSMA-mediated cytotoxicity [118,119]. Subsequent in vivo studies demonstrated potent anti-tumor activity in mouse xenograft models with human prostate and breast cancer cells, and importantly with much reduced systemic toxicity [118,119]. A Phase 1 clinical trial was completed in patients with refractory, advanced or metastatic solid tumors. G202 was found to be well tolerated in patients at doses up to 88 mg/m^2^ (or 2.4 mg/kg) and determined to have a favorable pharmacokinetic profile with a terminal half-life of 21 h, and distribution equivalent to plasma volume [123]. Several Phase 2 clinical trials have completed in prostate cancer, renal cell carcinoma, hepatocellular carcinoma, and glioblastoma but clinical results have not yet been reported [124].

### 4.4. Miscellaneous Peptide-Drug Conjugates

Doxorubicin has been chemically conjugated to a number of other peptides, including cell penetrating peptides (CPP) [125,126], tumor homing peptide Lyp-1 [127], RGD peptides [40], somatostatin [128,129,130], and bombesin/gastrin-releasing peptide (BN/GRP) [128,131,132], and broadly studied. BIM-23A760, a conjugate of somatostatin and dopamine is in clinical stage development for the treatment of pituitary adenomas [133,134,135]. A list of peptide-drug conjugates that have progressed into clinical development is summarized in Table 1. Given the sizable unmet medical need in many cancers, it has been the dominant disease for exploring the potential for selectively delivering toxic substances. Nonetheless, the potential for targeted therapy and synergistic efficacy between peptides and small molecules is clear and extends to forms of pharmacology beyond cytotoxicity. Recent applications of peptide-drug conjugates are emerging in other diseases areas, specifically cardiometabolic diseases where multi-mode pharmacology has been a traditional hallmark for successful disease management.

## 5. Representative Peptide-Drug Conjugates in Preclinical Space

### 5.1. GLP-1-Estrogen Conjugate

Estrogens are a group of steroid hormones which are commonly used in contraception and hormone replacement therapy. Estrogens also have notable beneficial effects on insulin signaling, glucose production, appetite, and energy expenditure to promote their potential use in the treatment of diabetes, obesity, and associated metabolic diseases [166,167,168,169]. However, the chronic use of estrogens has been complicated by oncogenic propensity in gynecological tissues and the increased risk for cardiovascular diseases (CVD) [170]. It has been suggested that tissue-targeted estrogens that selectively function in liver, adipose, pancreas, hypothalamus, but not in ovaries, uterus, and breast could prove efficacious and safe [171,172]. This has long been a priority in the search for small molecule selective estrogen receptor modulators [173,174].

**Figure 5:**
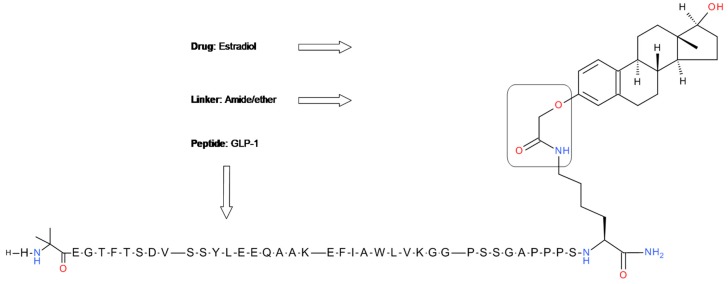
Chemical structure of GLP-1-estradiol conjugate.

The prospect of using a peptide hormone to target and supplement estrogen pharmacology was advanced by Finan et al. [175]. GLP-1 agonists have emerged as powerful therapy in the treatment of type 2 diabetes, obesity with proven CV benefits. It exerts its effects at specific receptors enriched in the endocrine pancreas and hypothalamic control centers of metabolism [176,177]. A GLP-1 estrogen conjugate formed by an ether link between 17β-estradiol and a lysine side chain amine at position 40 of GLP-1 was synthesized and evaluated in vitro and in vivo (Figure 5) [175]. This conjugate was fully active at the GLP-1 receptor in cell-based assays, and proteolytically stable in human plasma under physiological conditions for at least 120 h, reducing the prospect for premature plasma release. The conjugate demonstrated additive metabolic benefits of GLP-1 and estrogen to reverse obesity, hyperglycemia, and dyslipidemia in diet-induced obese (DIO) mice. Importantly there was no sign of estrogen associated gynecological toxicity or oncogenicity in the conjugate relative to what was observed in unstable conjugates that released systemically acting estrogen [175]. The anorexigenic effects of GLP-1 results from central action and the conjugate delivered estrogenic action to neurons in the dorsal raphe nuclei in female mice and suppressed binge-like eating behavior [178]. Further, it can activate both GLP-1 and estrogen receptors in the supra-mammillary nucleus in rats, resulting in superior effects on food intake and reward, to reduce body weight [179]. Moreover, the conjugate improves insulin sensitivity and glucose homeostasis in non-diabetic mice [180], attenuates hyperphagia and protects beta cell health in New Zealand Obese mice [181]. All these benefits were observed to be much superior to what GLP-1 alone or an untargeted combination provided. These studies demonstrate the enhanced therapeutic efficacy and safety of a GLP-1-estrogen conjugate, which justifies translational study in clinical diabetes and obesity. Similarly, a GLP-1-dexamethasone conjugate was also recently synthesized [182], and its combined therapeutic benefits characterized in metabolically compromised mice. Such a conjugate delivered potent effects in obese mice to lower body weight, improve glucose tolerance, and enhance insulin sensitivity with a reduction in hypothalamic and systemic inflammation. This conjugate was devoid of the adverse effects on glucose handling, bone and body weight typified by chronic systemic action of dexamethasone.

### 5.2. Glucagon-T3 Conjugate

Thyroid hormones are iodinated tyrosine-based amino acids produced by the thyroid gland and are widely prescribed for the treatment of thyroid hormone deficiency [183]. Thyroid hormones have profound effects on metabolism, increasing energy expenditure, fat oxidation, and cholesterol metabolism via multiple pathways to promote therapeutic potential in metabolic diseases [184,185]. However, like estrogens, thyroid hormones are associated with many adverse effects including increased heart rate, muscle wasting, and reduced bone density [186]. Liver-targeted thyromimetics have revealed that it is possible to impact hepatic lipids and atherogenic lipoproteins without the associated adverse effects [187]. Therefore, targeting thyroid hormone action to the liver and adipose tissues and away from the cardiovascular system might also constitute a viable approach to safely harness the metabolic benefits. Glucagon is a hormone well recognized as a counter-regulatory hormone to insulin in its hepatic action to stimulate glucose production. Less well appreciated are the other attributes of glucagon pharmacology which includes body weight lowering, lipid-lowering and cardiovascular protection [188]. These effects derive from the direct and indirect hepatic action of glucagon to promote lipolysis and thermogenesis. It was envisioned that conjugation of thyroid hormone and glucagon could complement one another in improving body weight while mitigating the ability of thyroid hormone to elevate plasma cholesterol [189].

**Figure 6:**
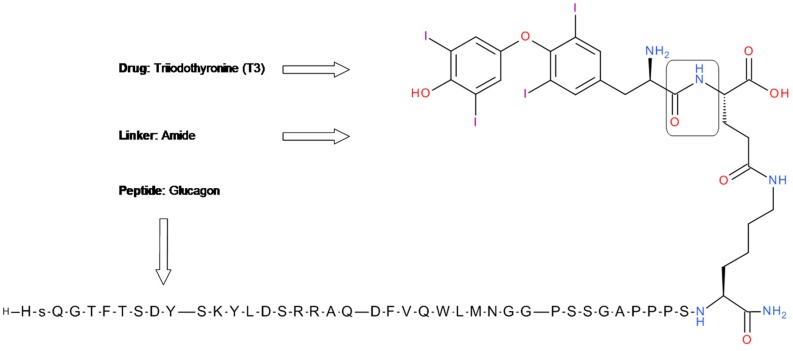
Chemical structure of glucagon-T3 conjugate.

A chemical conjugate of glucagon and the most bioactive form of thyroid hormone, 3,3,5-triiodothyronine (T3), was synthesized and biologically characterized (Figure 6) [189]. The conjugate has a DPP4 resistant d-serine at the second amino acid residue, a solubility enhancing eleven amino acid extension sequence at the C-terminus, and a gamma glutamic acid (gGlu) spacer linking the C-terminal lysine side chain amine and the carboxylate of T3. The conjugate preserved full glucagon in vitro potency at its receptor (EC_50_: 50 pM). The conjugate demonstrated dramatic metabolic benefits such as body weight lowering via increased energy expenditure, improved plasma cholesterol and triglyceride management, and much reduced hepatic liver stores in a mouse model of NASH [189]. The T3 was documented to be enriched in the liver, but not in pancreas or heart where glucagon receptor expression is less prominent. Analogous experiments in glucagon receptor knockout mice, as well as employment of peptide-conjugates devoid of one or the other hormonal activity, demonstrated that the metabolic benefits were the result of glucagon pharmacology and its targeting of thyroid hormone activity predominantly to the liver. Importantly, concurrent T3 activity counteracted the adverse diabetogenic effects of glucagon while glucagon lessened T3 elevation of cholesterol and its hepatic-targeting eliminated any apparent adverse cardiovascular or bone effects. Hence, pairing glucagon and thyroid hormones as a peptide-drug conjugate provides efficacious management of multiple elements in the metabolic syndrome, including hyperglycemia, obesity, fatty liver disease, and atherosclerosis.

### 5.3. Knotting Peptide Gemcitabine Conjugate

**Figure 7:**
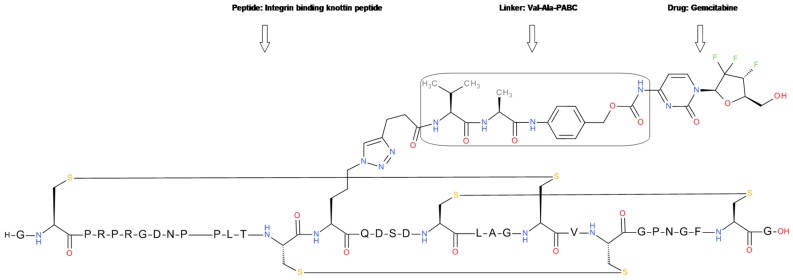
Chemical structure of knotting peptide ecballium elaterium trypsin inhibitor (EETI)-2.5Z-gemcitabine conjugate.

Integrins are a class of cell adhesion transmembrane receptors that regulate cell growth and function and are associated with several diseases including cancer, infection, and autoimmune diseases [190]. Integrin overexpression is linked to tumor proliferation and migration, which promotes disease progression and reduced patient survival [191,192]. Therefore, integrin antagonists are being clinically developed as therapeutics against cancer [193]. Integrins also provide opportunities for targeted peptide-drug conjugates. Several integrin targeting peptides were conjugated to cytotoxic agents for targeted tumor delivery. These include an RGD-doxorubicin conjugate, an RGD-Pt(iv) complex conjugate, and recently an integrin targeting knottin peptide gemcitabine conjugate [39,40,50,194]. Gemcitabine is a nucleoside based chemotherapeutic agent that blocks DNA replication and is used in the treatment of multiple cancers [195,196]. Like many other cancer drugs, gemcitabine is unrestricted in its action and can kill normal cells. To selectively target tumor cells, gemcitabine was conjugated to an integrin binding knottin peptide named ecballium elaterium trypsin inhibitor (EETI)-2.5Z. It has three intramolecular disulfide bonds to confer high thermal and proteolytic stability (Figure 7) [194]. EETI-2.5Z has low nanomolar binding affinity at integrins expressed on tumor cells, and conjugation with gemcitabine did not measurably affect its activity. Among various chemical conjugates that included an ester, carbamate, amide, and cathepsin B cleavable Val-Ala-PABC linkers, the EETI-2.5Z-Val-Ala-PABC-gemcitabine was observed to be highly stable in cell culture, with minimal premature drug release. More importantly, this conjugate exhibited very potent growth inhibition (ED_50_ of 1–10 nM) against a variety of cancer cells, including glioblastoma, breast, ovarian, and pancreatic cancer cells. The growth inhibition was abolished by the addition of excess unconjugated EETI-2.5Z, suggesting integrin-mediated internalization of gemcitabine pharmacology. PANC-1 pancreatic cancer cells have very high resistance to gemcitabine because of the diminished nucleoside transporter activity in these cells. Nonetheless, EETI-2.5Z-Val-Ala-PABC- gemcitabine was able to overcome the resistance and exhibited a 25-fold enhanced inhibitory activity relative to that of gemcitabine. Hence this peptide-drug conjugate further validated integrin as a therapeutic target for cancer and confirmed that peptides can successfully serve as an alternative to antibody targeted drug delivery. Of course, further preclinical animal studies and eventually human studies must be completed to prove its therapeutic efficacy and safety. It is noteworthy that gemcitabine has also been conjugated to a GnRH agonist [197], similar to the conjugate of GnRH with doxorubicin as discussed previously.

## 6. Linker and Conjugation Chemistry

**Figure 8:**
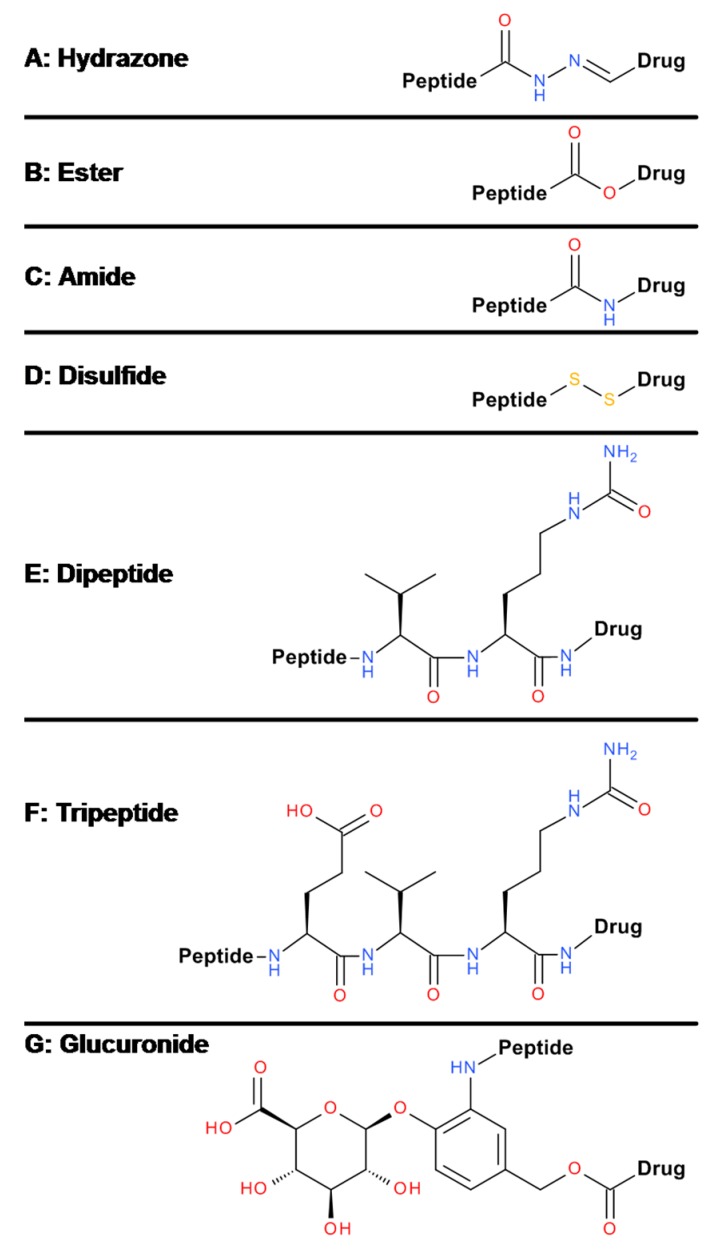
Linker technologies in peptide-drug conjugates.

The linker is a critical part of a peptide-drug conjugate that integrates the peptide and small molecule medicinal agents. The linker in concert with the peptide and the drug acts to maintain structural integrity during plasma circulation for a sufficient time and preventing premature release of the drug that might result in off-target adverse effects. Nonetheless, the linker should efficiently and specifically release the drug once tissue-targeted to enable a pharmacological effect. Linker technology has largely matured in the neighboring field of ADCs [198,199,200], where esters, amides, hydrazones, disulfides, and cathepsin B cleavable dipeptides have emerged as the preferred choices (Figure 8). This work has resulted in the development of three marketed drugs (Mylotarg, Adcetris, and Kadcyla), and a score of ADCs currently in clinical assessment [61,68,69,201,202,203,204]. These linkers and others have been extensively reviewed elsewhere as referenced. Briefly, hydrazones are relatively stable in plasma, but readily cleaved under acidic conditions, including endosomes and lysosomes where pH resides between 4.5–6.0. Ester bonds are widely used for conjugating drugs to peptides given their relatively straightforward synthesis and well-characterized cleavage by esterases, or under acidic conditions [198,199,200]. Carbamates perform similar to esters with comparable cleavage mechanisms, but typically with somewhat enhanced chemical and plasma stability. Although esters do not provide high plasma stability, it is still possible to successfully target oncolytic agents. If the goal is to develop a drug candidate of extended duration where the therapeutic index is appreciably increased, an amide bond may be preferable given the much-enhanced chemical and enzymatic plasma stability. Amide bonds are typically processed in lysosomes by multiple proteases to release the conjugated small molecules in a biologically active form [26,120,175]. If cleavage is not observed, dipeptide linkers such as Val-Cit and related dipeptides should be considered, as these can be cleaved by intracellular cathepsin B and other proteases [205]. A recent advance in peptide-based linkers combines Val-Cit with tertiary and heteroaryl amines to achieve traceless release [206]. This overcomes the challenges to employ tertiary amine bioactive molecules as payloads. The tripeptide linker Glu-Val-Cit was reported to further enhance stability and efficacy in mice when compared to the dipeptide linker Val-Cit [207]. It is known that Val-Cit linker, although stable in human plasma, is unstable in mouse plasma due to the cleavage by extracellular carboxylesterases, which causes translational inconsistency when comparing clinical and preclinical data. Thus, the amide bond seems to confer suitable chemical and plasma stability. Another enzyme cleavable linker is the β-glucuronide-based linker such as glucuronide-MABC, this linker offers benefits of high aqueous solubility, serum stability, and facile drug release. The cleavage is promoted by β-glucuronidase which is abundantly present in lysosomes and overexpressed in certain tumors [208,209]. Finally, disulfide bonds are extensively employed in peptide-drug conjugates, owing to what can be high plasma stability, and yet well-known intracellular cleavage by disulfide reduction. Their stability can be further enhanced through the addition of one or two methyl groups adjacent to the disulfide bond. Hence, hydrazone, ester, amide, disulfide, dipeptide, tripeptide, and glucuronide-based linkers provide a diverse set of linkers that can meet most needs in the assembly of peptide-drug conjugates for targeted delivery (Figure 8).

In ADCs, the linker is installed by conjugation of the linker-drug moiety to the antibody which most often occurs on the reactive surface residues of antibodies such as cysteine and lysine residues [68,200,210,211,212,213]. The conjugation is a very critical step in ADC synthesis since typically there are multiple cysteine and lysine residues and without optimization results in a heterogeneous distribution in site and number of drugs that are loaded to the antibody. Molecular engineering and enzymatic modification have provided non-native amino acids to facilitate site-specific conjugation, but there remain technical challenges in commercial-scale production of homogenous ADCs [60,214,215,216]. Peptide-drug conjugates benefit in employing total chemical synthesis that involves orthogonal side chain protection to control product integrity. Appropriately functionalized amino acids (natural and non-natural) can be utilized for conjugation and examples include alkylation [217,218,219], Suzuki coupling [220,221], Glaser reaction [222], Diels-Alder reaction [223], CH activation [224], and oxime ligation [225,226]. The single requirement is that the peptide, the small molecule and the conjugated products are chemically stable under the conditions for synthesis and purification. In practice, most of the conjugation reactions still employ a cysteine and lysine residue, but with methods that govern selective modification. Additionally, click-based conjugation has been widely used given its orthogonal and relatively mild reaction conditions [227,228].

### 6.1. Amide Bond Formation

**Scheme 1:**
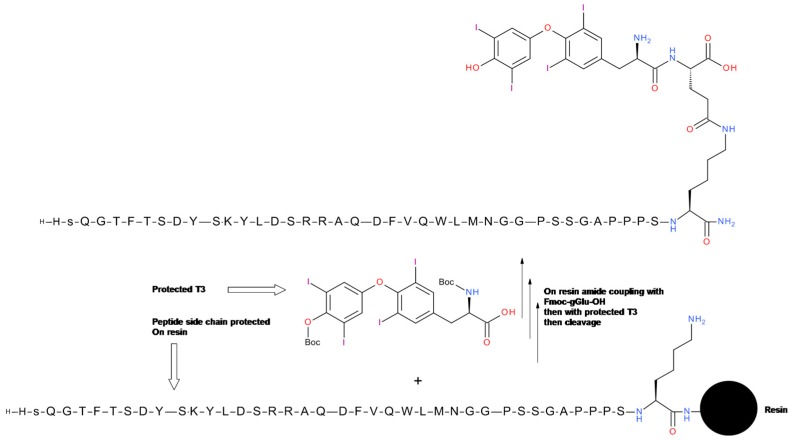
Conjugation of glucagon and T3 via an amide bond. Amide bond was formed between T3 and protected peptide glucagon with a spacer gGlu on resin [189].

**Scheme 2:**
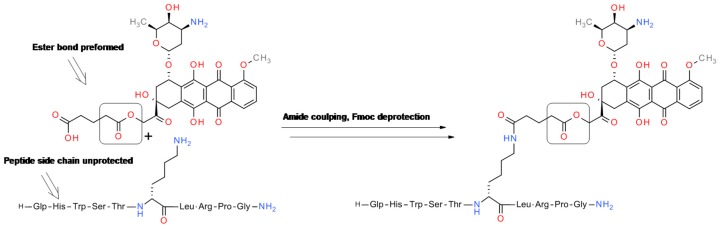
Conjugation of GnRH peptide and doxorubicin (Zoptarelin Doxorubicin, AN-152, AEZS-108). Amide bond was formed between doxorubicin and unprotected peptide GnRH [234].

Amide bond formation is the most straightforward way to attach drug molecules to peptides [1,2,3,4,50,51]. In an Fmoc-based peptide assembly on a solid support, a lysine is typically orthogonally protected with Mtt. This protecting group once selectively removed can be coupled to a carboxylic acid of amino acid or small molecule under standard peptide coupling protocols [229,230]. This approach is demonstrated in the synthesis of glucagon-T3, where selectively de-protected lysine side chain amino at position 40 on glucagon backbone first reacted with amino acid Fmoc-gGlu-OH, and then with Boc protected T3 (Scheme 1) [189]. The carboxylic acids of protected T3 could also be pre-activated to something such as a succinate ester to allow direct reaction with the amine without coupling reagents [213,231,232,233]. Obviously, the linker and drug molecules must tolerate the conditions employed in peptide-resin cleavage (95% TFA with 2.5% TIS, and 2.5% water). Alternatively, selective conjugation can occur with unprotected peptides, employing the enhanced nucleophilic nature of the ε-amino group, as shown in the synthesis of GnRH-doxorubicin conjugate in Scheme 2 [234]. A dipeptide Val-Cit/Ala linker can be inserted using a similar synthetic approach, and an acidic acid such as Asp with orthogonal protection can also be used to conjugate with amine-containing drugs [7,235,236]. In summary, an amide bond is the most conventional and versatile approach to peptide-drug conjugates of suitable chemical and biological stability.

### 6.2. Disulfide Bond Formation

**Scheme 3:**
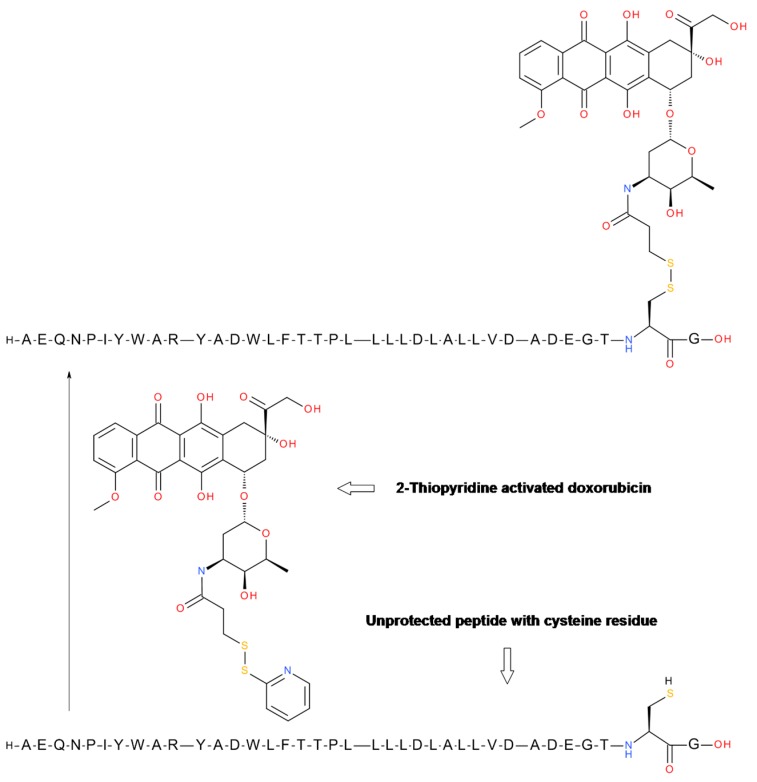
Conjugation of pH low insertion peptide (pHLIP) and doxorubicin via a disulfide bond. Disulfide bond was formed between 2-thiopyridine activated doxorubicin and unprotected peptide pHLIP via a cysteine residue [238].

Disulfide bonds have been extensively used in the conjugation of peptides to drugs given the selective nature in formation and the intracellular reduction-mediated release of the drug [210,211,213,237]. Typically, a cysteine residue or a similar thiol is introduced to a noncritical region of the peptide, which is subsequently coupled through a sulfhydryl pre-activated drug (via 2-thiopyridine or DTNP). This reaction often proceeds very quickly and selectively to yield conjugated drug-products (Scheme 3) [238]. The inverse approach is also commonly employed where a peptide cysteine once activated is coupled to a thiol-containing drug to form a conjugated drug-product. There are also several other ways to form a disulfide bond if the more common methods fail, which collectively constitute a diverse set of reactions to construct disulfide bonds [239,240]. Relative to amide bond formation the increased orthogonality of cysteine lessens the need to introduce the drug to a protected peptide, which constitutes a sizable advantage when there are lysines and when the small molecule drug is not suitable for use in standard peptide synthetic protocols. It is worth noting that synthesis of disulfide bonds with adjacent gem-dimethyl groups are better achieved by pre-activating the gem-dimethyl containing sulfhydryl group to react with the less hindered sulfhydryl group. This is a consequence of steric hinderance that serves to reduce the reactivity of sulfhydryl groups surrounded by a gem-dimethyl group.

### 6.3. Thioether Formation

**Scheme 4:**
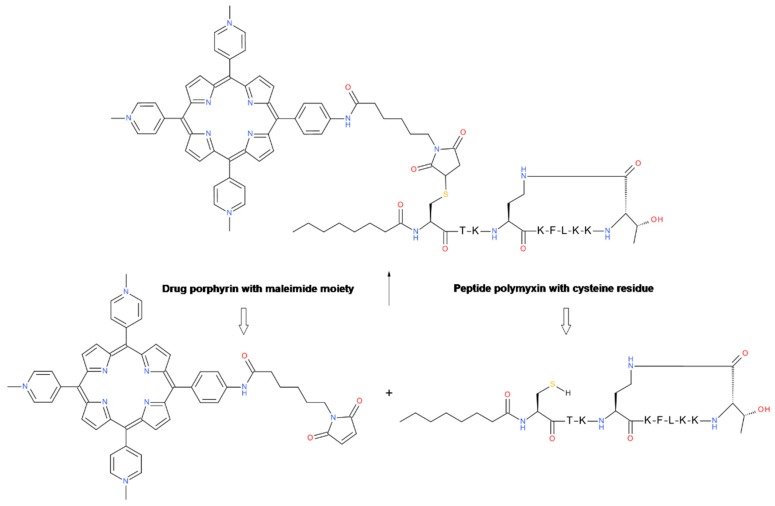
Conjugation of Polymyxin B and antimicrobial porphyrin via a thiol-maleimide bond. Thiol-maleimide bond was formed between maleimide containing porphyrin and unprotected peptide polymyxin B with a cysteine residue [241].

The thiol-maleimide reaction is a widely used method to conjugate peptides and drugs, where a peptide sulfhydryl group reacts with a pre-installed maleimide group on the drug (Scheme 4) [201,203,204,241,242,243]. The reaction is selective and efficient across a variety of solvents across a wide pH range, and the thioether bond offers reasonable chemical stability. Given the Michael reaction mechanism, the product is reversed under alkaline conditions and thiol exchange is reported in storage or in the presence of serum [60,216,244]. This serves to shorten the drug product shelf-life and often the circulating half-life in plasma. Recently, a ring opening stabilization strategy [245,246,247] or next-generation maleimide (NGM) strategy [248,249,250] was reported to convert the thiol-maleimide bond to a more stable thioether. In addition to the thiol-maleimide reaction, thiol alkylation is also commonly utilized to form thioether conjugated peptides and drugs. Peptide cysteine residues are typically alkylated through a bromo or iodo acetamide group in the drug, or vice versa [210,211,217,218,219]. Given the higher reactivity of cysteine towards these alkylating groups under slightly basic conditions, there is no competing amine alkylation, which makes it highly useful in the assembly of chemically and biologically stable peptide-drug conjugates.

### 6.4. Click Reaction

**Scheme 5:**
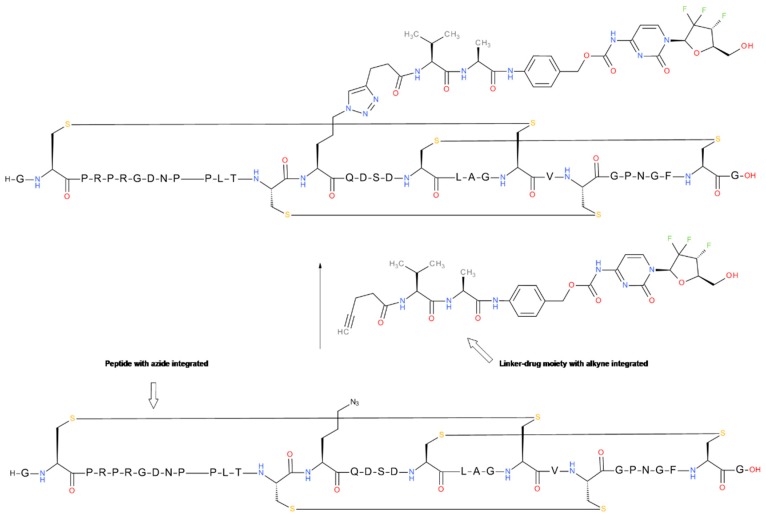
Conjugation of knotting peptide and gemcitabine via click chemistry. The triazole was formed between the alkyne-containing gemcitabine and an unprotected azide-containing knotting peptide [194].

The so-called click reaction between an alkyne and azide (1,3 dipolar cycloaddition) is a widely used method for bioconjugation that is independent of cysteine and lysine residues [214,227,228]. This is a very attractive approach for connecting peptides and drugs when there are multiple lysine or cysteine residues in the peptide. The reaction occurs under mild conditions. It is efficient and devoid of any cross-reaction with natural amino acids, making it an excellent method to make homogenous peptide-drug conjugates. An example is shown in Scheme 5, where the azide group is stable in conventional methods of peptide synthesis, cleavage, and purification. The alkyne group is incorporated into the drug with an additional linker, and the peptide and drug fragments are joined together under a classical Cu-catalyzed reaction. Recent applications of click reaction in peptide-drug conjugate synthesis include EphA2-paclitaxel, GLP-1-vitamin B12, and peptide-glycolipid [251,252,253,254,255].

## 7. Peptide-Drug Conjugate Design Considerations

It is relatively straightforward to make peptide-drug conjugates, given the established synthetic conjugation strategies but there are a few central considerations that must be addressed to enhance the chance for pharmacological success. First and foremost, there must be a strong biological basis for the specific combination of the two molecular entities that compose the conjugate. Ideally, the drug and peptide operate biologically on different pathways that complement and better yet synergistically provide superior therapeutic outcomes than either operating alone. This is observed in a conjugate such as Zoptarelin Doxorubicin where GnRH functions in androgen deprivation therapy (ADT) for prostate cancer and doxorubicin is a proven oncolytic for multiple cancers. A second consideration pertains to drug targeting, where small molecule-based agents are inherently efficacious but with liabilities usually pertaining to toxicity resulting from systemic exposure. Conjugation to a suitable peptide can provide tissue-specific delivery to concentrate the pharmacology at a preferred site and lessen off-target adverse effects. This is exemplified by the somatostatin conjugate with 177Lu (177Lu-Dotatate). A third consideration is the efficiency in drug transport as peptides typically exhibit therapeutic effects at an extracellular target receptor, and a small molecule drug via an intracellular target. The conjugation targets the small molecule but places a restriction in its performance that is dependent upon the peptide-mediated internalization and subsequent intracellular release. The extracellular peptide receptor must be of sufficient capacity to internalize enough small molecule in a ligand-dependent manner for the drug to render a pharmacological effect. Peptide receptor agonists are more likely to fulfill this requirement as they are proficiently internalized and return to the plasma membrane for reuse in a manner that is more certain than peptide-based antagonists. A fourth consideration pertains to potency and the need to align them across the delivery peptide and small molecule drug. Peptide agonists are often very potent molecules operating at nanomolar or lower concentrations at their target receptors and small molecule drugs are often challenged to match this inherent potency. Increasing the drug payload by a stoichiometric ratio relative to the peptide is one way to achieve potency alignment, but this requires a minimal potency difference in the constituents since there is a practical limit to the molar equivalents of a small molecule that can be attached to a peptide before it loses its biological and physical properties. Increasing the dose to levels beyond that which is necessary for full peptide agonism may be possible if the peptide is devoid of adverse effects when used at super-pharmacological levels. This is a property that is specific to each peptide, as some such as insulin have a narrow therapeutic index with life-threatening consequences for overdosing, while others are more forgiving. Lastly, many small molecules have an appreciable affinity towards plasma proteins such as albumin and conjugation to a peptide can significantly alter the pharmacokinetic profile. This effect can alter the biology of the peptide or incorrectly suggest that an altered activity is a function of the combined biology of the drug candidate. PTH is a hormone where it is well appreciated that pulsatile administration can potently build bone mass and strength, while sustained delivery is known to be bone catabolic. Consequently, it would be a dangerous targeting peptide for any small molecule that alters its pharmacokinetics, independent of any direct change in its interaction with a target receptor. Similarly, nuclear hormones constitute excellent small molecules for tissue targeting but often possess high-affinity plasma protein binding and as such, any peptide conjugate needs to be shown unaltered time-action to attribute additive biology from two supplemental pharmacological mechanisms. In summary, the synthetic chemistry is relatively straightforward, but the design considerations are of utmost importance in selecting matching pairs that are capable of providing supplemental efficacy and selectivity.

## 8. Outlook and Perspective

Peptide-drug conjugates are a unique class of molecules that integrate peptides and small molecule drugs to achieve increased therapeutic outcomes. They represent an important field in drug discovery that is related in principle to antibody-directed drug targeting. These molecular conjugates leverage the inherent and unique pharmacological abilities of the peptide and the small molecule. A variety of conjugates have been discovered and developed in several therapeutic areas including numerous diseases of endocrine, infectious, and autoimmune origins. Several drug candidates have demonstrated promising preclinical results, and a few have progressed to registered medicines, most notably in the treatment of various cancers. The most powerful examples are those where the macromolecular native of the peptide is used to target small molecule effects to those tissues where the peptide is biologically active. In such instances where the peptide provides supplemental pharmacology to the small molecule the biological outcomes are enhanced, and often with far less off-target toxicity. In addition, conjugation to peptides can diminish common physical challenges in the development of small molecule drugs, and in particular, those pertaining to high lipophilicity, poor solubility and cell impermeability. The recent regulatory approval for GHIH-177Lu conjugate (177Lu-Dotatate) is a notable example of success and provides strong momentum for further applications. The research pertaining to tissue-specific delivery of nuclear hormones in the treatment of the metabolic syndrome broadens the conceptual approach beyond the delivery of cytotoxic agents to achieve mixed-mode agonism of small and large pharmacophores with different mechanisms of action.

Nevertheless, sizable challenges remain and there have been more failures than successes. The obstacles are numerous, and they pertain to both biological and chemical aspects of the strategy. The latter seems more manageable and to a finite degree are related to the biological uncertainties. Specificity remains an elusive goal as it is a rare occurrence when an extracellular target is found at only a single tissue rendering the approach more suitable for improving therapeutic index than engendering absolute specificity. This is a great obstacle when the objective is the elimination of metastatic disease as the destruction of the last percent or less of diseased cells requires dose intensity that leads to toxicity in unintended tissues. In contrast, if the therapeutic objective is to enhance the therapeutic index by an order of magnitude to permit increased dosing by tenfold than this is something more easily achieved by enriching the pharmacological action at certain tissues. The second challenge of appreciable complexity is the immature nature of the collective knowledge of intracellular biochemistry. While it is clear that peptide cycling-receptors can internalize a ligand that carries a small molecule pharmacophore it is less clear how to transport the entity to the intracellular site where biological action occurs. The escape of small molecules from the endosome remains an emerging field of study and once liberated facilitated transport to preferred intracellular locations such as the mitochondria, nucleus and other sites largely remains an unknown. Furthermore, once a desired biological effect is achieved the question of its termination stands tall and in particular the danger for reverse extracellular transport to sites that were purposely avoided in peptide-directed tissue targeting. The question is whether the primarily targeted tissues are capable of metabolizing the small molecule drug to something that is innocuous to other tissues once released to general circulation? Finally, there remains the common imbalance between the inherent potency of peptides and small molecules, such that there is a huge deficiency in the transport capacity of the macromolecule relative to the required concentration of the small molecule. In this regard, the assembly of defined macromolecular complexes seems the best hope where large amounts of a single substance or even more than one substance can be packaged for targeted delivery in a nanoparticular or exosome by a peptide-based surface ligand. It requires additional refinement to avoid the endogenous defense mechanisms that are designed for non-specific clearance of macromolecular biological and synthetic entities. The speed in which these fundamental challenges are addressed will to a large degree determine the productivity in this molecular design and the fate of future peptide-drug candidates. Until that point when the molecular design is better defined individual drug candidates will continue to emerge by successfully circumnavigating the current obstacles. We remain sanguine about the amount of research that remains to be performed and its ability to further advance the field.

## Figures and Tables

**Table 1:** Peptide-drug conjugates in various clinical development stages.

Generic Name	Indication	Peptide	Drug	Linker	Mechanism	Status	Reference
Lu177-dotatate	Dastroenteropancreatic neuroendocrine tumors	Somatostatin analogue Octreotide	Radio therapeutic agent Lu177	Amide (Lu177 chelating to metalchelating molecule DOTA)	Somatostatin receptor 2 SSTR2 mediated delivery of nucleotide 177Lu	Approved by FDA and EMA	[76,85,86,87,88,89,90,91,92,93,94,95,96,97,98,99]
[111In-DTPA-D-Phe1]-octreotide	Imaging/diagnostic	Somatostatin analogue Octreotide	Radio therapeutic agent 111In	Amide (111In chelating to metalchelating molecule DOTA)	Somatostatin receptor 2 SSTR2 mediated tumor scintigraphic imaging	Phase 1 completed	[136,137]
Zoptarelin Doxorubicin, AN-152, AEZS-108	Endometrial cancer Ovarian cancer	GnRH/LHRH	Doxorubicin	Ester	GnRH mediated delivery of doxorubicin to cancer cells	Phase 3 completed	[96,98,99,100,101,102,103]
ANG1005 GRN1005	Metastases brain cancer	Angiopep-2	Paclitaxel	Ester	Low-density lipoprotein receptor-related protein 1 (LPR1) mediated brain uptake	Orphan drug for glioblastoma multiform, Several phase 2 ongoing	[109,110,111,112,113,114,115,116]
Mipsagargin G202	Various Cancer	Tetrapeptide	Thapsigargin	Ester	Extracellularly tumor-activated prodrug of Thapsigargin	Phase 2 completed	[118,119,120,123]
Paclitaxel poliglumex CT2103	Various cancer	Poliglumex	Paclitaxel	Ester	Enhanced permeability of tumor vasculature and lack of lymphatic drainage prolonged tumor exposure to the active drug while minimizing systemic exposure	Phase 3 completed	[138,139,140,141,142]
EP-100	Cancer	GnRH/LHRH	CLIP71	Amide	GnRH receptor-mediated cancer cell membrane lysis	Phase 2 completed	[143,144,145]
BIM-23A760	Pituitary adenomas	Somatostatin	Dopamine	Amide/Thioether	Somatostatin/dopaminethe dual action inhibit the expression/secretion of several pituitary hormones (especially GH/PRL)	Phase 2 terminated	[133,134,135]
CGC 1072	Psoriasis	Heptaarginine	Cyclosporin A	Ester	CPP mediated topical delivery and inhibition of inflammation	Phase 2, discontinued	[146,147]
KAI-1455	Ischemic organ injury	TAT47-57	εPKC activator	Disulfide	CPP mediated εPKC activator delivery	Phase 1	[148]
KAI-1678	Neuropathic and inflammatory pain	TAT47-57	δ-Protein kinase C inhibitor peptide	Disulfide	CPP mediated εPKC inhibitor delivery	Phase 2 completed	[149,150,151]
KAI-9803	Myocardial infarction & Cardiovascular disease	TAT47-57	δ-Protein kinase C inhibitor peptide	Disulfide	CPP mediated εPKC inhibitor delivery to reduce the injury to myocardial and endothelial cells during a heart attack	Phase 2 completed	[152,153,154]
XG-102	Post-cataract surgery, intraocular inflammation and Pain	Tat48-57	31-mer peptide JNK inhibitor	Disulfide	CPP mediated JNK inhibitor delivery to reduce pain and inflammation upon cataract surgery	Phase 3 completed	[155,156,157,158]
DTS-108	Cancer	DPV1047 Vectocell peptide	SN38	Ester	CPP DPV1047 mediated delivery of chemotherapeutic drug SN38	Phase 1 completed	[159,160]
DTS-201	Cancer	Tetra peptide	Doxorubicin	Amide	Extracellularly tumor-activated prodrug of doxorubicin	Phase 2 completed	[161,162,163]
BT-1718	Cancer	Bicyclic peptide	Maytansinoid	Disulfide	Membrane type 1-matrixmetalloprotease mediated toxin delivery	Phase 1	[164]
177Lu- PSMA-617	Cancer	Glutamate-urea-lysine	Radio therapeutic agent Lu177	Amide (Lu177 chelating to metalchelating molecule DOTA)	Prostate-specific membrane antigen (PSMA) mediated delivery of nucleotide 177Lu	Phase 3	[165]

## Abbreviations

ADCs	Antibody-Drug Conjugates
ADT	Androgen Deprivation Therapy
Ala	Alanine
BBB	Blood–Bain–Barrier
Cit	Citrulline
DAR	Drug-Antibody Ratio
DDI	Drug-Drug Interactions
DIO	Diet-Induced Obese
DOTA	1,4,7,10-Tetraazacyclododeane
DPP4	Dipeptidyl Peptidase 4
DTNP	5,5′-Disulfanediylbis(2-nitrobenzoic acid) or Ellman’s reagent
GHIH	Growth Hormone-Inhibiting Hormone
GIP	Gastric Inhibitory Polypeptide
GLP-1	Glucagon-Like Peptide 1
GLP-2	Glucagon-Like Peptide 2
gGlu	gamma glutamic acid
GnRH	Gonadotropin-Releasing Hormone
LHRH	Luteinizing Hormone-Releasing Hormone
LRP1	Low-Density Lipoprotein Receptor-Related Protein 1
NSCLC	Non-Small Cell Lung Cancer
PABC	p-Aminobenzyl Carbamate
PRRT	Peptide Receptor Radionuclide Therapy
PSMA	Prostate Specific Membrane Antigen
PTH	Parathyroid Hormone
SERCA	the Sarco/Endoplasmic Reticulum Calcium ATPase
SSTR	Somatostatin Receptor

## References

[1] Henninot A., Collins J.C., Nuss J.M. (2018). The Current State of Peptide Drug Discovery: Back to the Future?. J. Med. Chem..

[2] Lau J.L., Dunn M.K. (2017). Therapeutic peptides: Historical perspectives, current development trends, and future directions. Bioorg. Med. Chem..

[3] Fosgerau K., Hoffmann T. (2015). Peptide therapeutics: Current status and future directions. Drug Discov. Today.

[4] Kaspar A.A., Reichert J.M. (2013). Future directions for peptide therapeutics development. Drug Discov. Today.

[5] Liu F., Li P., Gelfanov V., Mayer J., DiMarchi R. (2017). Synthetic Advances in Insulin-like Peptides Enable Novel Bioactivity. Acc. Chem. Res..

[6] Mijalis A.J., Thomas D.A., Simon M.D., Adamo A., Beaumont R., Jensen K.F., Pentelute B.L. (2017). A fully automated flow-based approach for accelerated peptide synthesis. Nat. Chem. Biol..

[7] Behrendt R., White P., Offer J. (2016). Advances in Fmoc solid-phase peptide synthesis. J. Pept. Sci..

[8] Lau J., Bloch P., Schaffer L., Pettersson I., Spetzler J., Kofoed J., Madsen K., Knudsen L.B., McGuire J., Steensgaard D.B. (2015). Discovery of the Once-Weekly Glucagon-Like Peptide-1 (GLP-1) Analogue Semaglutide. J. Med. Chem..

[9] Made V., Els-Heindl S., Beck-Sickinger A.G. (2014). Automated solid-phase peptide synthesis to obtain therapeutic peptides. Beilstein J. Org. Chem..

[10] Mitchell A.R. (2008). Bruce Merrifield and solid-phase peptide synthesis: A historical assessment. Biopolymers.

[11] Baeshen N.A., Baeshen M.N., Sheikh A., Bora R.S., Ahmed M.M., Ramadan H.A., Saini K.S., Redwan E.M. (2014). Cell factories for insulin production. Microb. Cell Fact..

[12] Meehl M.A., Stadheim T.A. (2014). Biopharmaceutical discovery and production in yeast. Curr. Opin. Biotechnol..

[13] Thayer A.M. (2011). Making Peptides At Large Scale. Chem. Eng. News.

[14] Mayer J.P., Zhang F., DiMarchi R.D. (2007). Insulin structure and function. Biopolymers.

[15] Blazynski C. (2017). 2016 Completed Clinical Trials: Industry Strategies Revealed and Graded.

[16] Waring M.J., Arrowsmith J., Leach A.R., Leeson P.D., Mandrell S., Owen R.M., Pairaudeau G., Pennie W.D., Pickett S.D., Wang J. (2015). An analysis of the attrition of drug candidates from four major pharmaceutical companies. Nat. Rev. Drug Discov..

[17] Thomas D.W., Burns J., Audette J., Carroll A., Dow-Hygelund C., Hay M. (2016). Clinical Development Success Rates 2006–2015.

[18] Wirtz V., Knox R., Cao C., Mehrtash H., Posner N.W., McClenathan J. (2016). Insulin Market Profile.

[19] (2018). Novo Nordisk Annual Report 2017.

[20] Moya-Garcia A., Adeyelu T., Kruger F.A., Dawson N.L., Lees J.G., Overington J.P., Orengo C., Ranea J.A.G. (2017). Structural and Functional View of Polypharmacology. Sci. Rep..

[21] Anighoro A., Bajorath J., Rastelli G. (2014). Polypharmacology: Challenges and opportunities in drug discovery. J. Med. Chem..

[22] Reddy A.S., Zhang S. (2013). Polypharmacology: Drug discovery for the future. Expert Rev. Clin. Pharm..

[23] Tschop M., DiMarchi R. (2017). Single-Molecule Combinatorial Therapeutics for Treating Obesity and Diabetes. Diabetes.

[24] Khajavi N., Biebermann H., Tschop M., DiMarchi R. (2017). Treatment of Diabetes and Obesity by Rationally Designed Peptide Agonists Functioning at Multiple Metabolic Receptors. Endocr. Dev..

[25] Sadry S.A., Drucker D.J. (2013). Emerging combinatorial hormone therapies for the treatment of obesity and T2DM. Nat. Rev. Endocrinol..

[26] Finan B., Yang B., Ottaway N., Smiley D.L., Ma T., Clemmensen C., Chabenne J., Zhang L., Habegger K.M., Fischer K. (2015). A rationally designed monomeric peptide triagonist corrects obesity and diabetes in rodents. Nat. Med..

[27] Demartis A., Lahm A., Tomei L., Beghetto E., Di Biasio V., Orvieto F., Frattolillo F., Carrington P.E., Mumick S., Hawes B. (2018). Polypharmacy through Phage Display: Selection of Glucagon and GLP-1 Receptor Co-agonists from a Phage-Displayed Peptide Library. Sci. Rep..

[28] Jall S., Sachs S., Clemmensen C., Finan B., Neff F., DiMarchi R.D., Tschop M.H., Muller T.D., Hofmann S.M. (2017). Monomeric GLP-1/GIP/glucagon triagonism corrects obesity, hepatosteatosis, and dyslipidemia in female mice. Mol. Metab..

[29] Gault V.A., Bhat V.K., Irwin N., Flatt P.R. (2013). A novel glucagon-like peptide-1 (GLP-1)/glucagon hybrid peptide with triple-acting agonist activity at glucose-dependent insulinotropic polypeptide, GLP-1, and glucagon receptors and therapeutic potential in high fat-fed mice. J. Biol. Chem..

[30] Pocai A., Carrington P.E., Adams J.R., Wright M., Eiermann G., Zhu L., Du X., Petrov A., Lassman M.E., Jiang G. (2009). Glucagon-like peptide 1/glucagon receptor dual agonism reverses obesity in mice. Diabetes.

[31] Day J.W., Ottaway N., Patterson J.T., Gelfanov V., Smiley D., Gidda J., Findeisen H., Bruemmer D., Drucker D.J., Chaudhary N. (2009). A new glucagon and GLP-1 co-agonist eliminates obesity in rodents. Nat. Chem. Biol..

[32] Andreassen K.V., Feigh M., Hjuler S.T., Gydesen S., Henriksen J.E., Beck-Nielsen H., Christiansen C., Karsdal M.A., Henriksen K. (2014). A novel oral dual amylin and calcitonin receptor agonist (KBP-042) exerts antiobesity and antidiabetic effects in rats. Am. J. Physiol. Endocrinol. Metab..

[33] Gydesen S., Andreassen K.V., Hjuler S.T., Hellgren L.I., Karsdal M.A., Henriksen K. (2017). Optimization of tolerability and efficacy of the novel dual amylin and calcitonin receptor agonist KBP-089 through dose escalation and combination with a GLP-1 analog. Am. J. Physiol. Endocrinol. Metab..

[34] Gydesen S., Hjuler S.T., Freving Z., Andreassen K.V., Sonne N., Hellgren L.I., Karsdal M.A., Henriksen K. (2017). A novel dual amylin and calcitonin receptor agonist, KBP-089, induces weight loss through a reduction in fat, but not lean mass, while improving food preference. Br. J. Pharm..

[35] Hjuler S.T., Gydesen S., Andreassen K.V., Karsdal M.A., Henriksen K. (2017). The Dual Amylin- and Calcitonin-Receptor Agonist KBP-042 Works as Adjunct to Metformin on Fasting Hyperglycemia and HbA1c in a Rat Model of Type 2 Diabetes. J. Pharm. Exp..

[36] Mullard A. (2017). Bispecific antibody pipeline moves beyond oncology. Nat. Rev. Drug Discov..

[37] Clarke S.C., Ma B., Trinklein N.D., Schellenberger U., Osborn M.J., Ouisse L.H., Boudreau A., Davison L.M., Harris K.E., Ugamraj H.S. (2018). Multispecific Antibody Development Platform Based on Human Heavy Chain Antibodies. Front. Immunol..

[38] Sedykh S.E., Prinz V.V., Buneva V.N., Nevinsky G.A. (2018). Bispecific antibodies: Design, therapy, perspectives. Drug Des. Dev..

[39] Ma L., Wang C., He Z., Cheng B., Zheng L., Huang K. (2017). Peptide-Drug Conjugate: A Novel Drug Design Approach. Curr. Med. Chem..

[40] Gilad Y., Firer M., Gellerman G. (2016). Recent Innovations in Peptide Based Targeted Drug Delivery to Cancer Cells. Biomedicines.

[41] Firer M.A., Gellerman G. (2012). Targeted drug delivery for cancer therapy: The other side of antibodies. J. Hematol. Oncol..

[42] Zagorodko O., Arroyo-Crespo J.J., Nebot V.J., Vicent M.J. (2017). Polypeptide-Based Conjugates as Therapeutics: Opportunities and Challenges. Macromol. Biosci..

[43] Qi Y., Chilkoti A. (2015). Protein-polymer conjugation-moving beyond PEGylation. Curr. Opin. Chem. Biol..

[44] Vhora I., Patil S., Bhatt P., Misra A. (2015). Protein- and Peptide-drug conjugates: An emerging drug delivery technology. Adv. Protein Chem. Struct. Biol..

[45] Staderini M., Megia-Fernandez A., Dhaliwal K., Bradley M. (2017). Peptides for optical medical imaging and steps towards therapy. Bioorg. Med. Chem..

[46] Albada B., Metzler-Nolte N. (2017). Highly Potent Antibacterial Organometallic Peptide Conjugates. Acc. Chem. Res..

[47] Santos R., Ursu O., Gaulton A., Bento A.P., Donadi R.S., Bologa C.G., Karlsson A., Al-Lazikani B., Hersey A., Oprea T.I. (2017). A comprehensive map of molecular drug targets. Nat. Rev. Drug Discov..

[48] Agarwal P., Sanseau P., Cardon L.R. (2013). Novelty in the target landscape of the pharmaceutical industry. Nat. Rev. Drug Discov..

[49] Smietana K., Siatkowski M., Moller M. (2016). Trends in clinical success rates. Nat. Rev. Drug Discov..

[50] Bohme D., Beck-Sickinger A.G. (2015). Controlling Toxicity of Peptide–Drug Conjugates by Different Chemical Linker Structures. ChemMedChem..

[51] Diaz D., Ford K.A., Hartley D.P., Harstad E.B., Cain G.R., Achilles-Poon K., Nguyen T., Peng J., Zheng Z., Merchant M. (2013). Pharmacokinetic drivers of toxicity for basic molecules: Strategy to lower pKa results in decreased tissue exposure and toxicity for a small molecule Met inhibitor. Toxicol. Appl. Pharm..

[52] Palleria C., Di Paolo A., Giofre C., Caglioti C., Leuzzi G., Siniscalchi A., De Sarro G., Gallelli L. (2013). Pharmacokinetic drug-drug interaction and their implication in clinical management. J. Res. Med. Sci..

[53] Ehrlich P. (1906). The relationship existing between chemical constitution, distribution and pharmacological action. Collected Studies on Immunity.

[54] Ehrlich P., Himmelwiet F. (1913). Chemotherapy. Proceedings of 17th International Congress of Medicine, in Collected Papers of Paul Ehrlich.

[55] Mathe G., Loc T.B., Bernard J.C.C. (1958). Effet sur la leucemie L1210 de la souris d’une combinaison par diazotation d’A-methopterine et de gamma-globulines de hamsters porteur de cette leucemie par heterogreffe. C. R. Acad. Sci..

[56] Ghose T., Cerini M., Carter M., Nairn R.C. (1967). Immunoradioactive agent against cancer. Br. Med. J..

[57] Ghose T., Nigam S.P. (1972). Antibody as carrier of chlorambucil. Cancer.

[58] Rowland G.F., O’Neill G.J., Davies D.A. (1975). Suppression of tumour growth in mice by a drug-antibody conjugate using a novel approach to linkage. Nature.

[59] Ford C.H., Newman C.E., Johnson J.R., Woodhouse C.S., Reeder T.A., Rowland G.F., Simmonds R.G. (1983). Localisation and toxicity study of a vindesine-anti-CEA conjugate in patients with advanced cancer. Br. J. Cancer.

[60] Beck A., Goetsch L., Dumontet C., Corvaia N. (2017). Strategies and challenges for the next generation of antibody-drug conjugates. Nat. Rev. Drug Discov..

[61] Perez H.L., Cardarelli P.M., Deshpande S., Gangwar S., Schroeder G.M., Vite G.D., Borzilleri R.M. (2014). Antibody-drug conjugates: Current status and future directions. Drug Discov. Today.

[62] Senter P.D., Sievers E.L. (2012). The discovery and development of brentuximab vedotin for use in relapsed Hodgkin lymphoma and systemic anaplastic large cell lymphoma. Nat. Biotechnol..

[63] Younes A., Yasothan U., Kirkpatrick P. (2012). Brentuximab vedotin. Nat. Rev. Drug Discov..

[64] Lambert J.M., Chari R.V. (2014). Ado-trastuzumab Emtansine (T-DM1): An antibody-drug conjugate (ADC) for HER2-positive breast cancer. J. Med. Chem..

[65] https://www.reuters.com/article/us-pfizer-mylotarg/pfizer-pulls-leukemia-drug-from-u-s-market-idUSTRE65K5QG20100621.

[66] https://www.fda.gov/drugs/informationondrugs/approveddrugs/ucm572133.htm.

[67] https://clinicaltrials.gov/ct2/results?intr=Antibody-Drug+Conjugate&Search=Apply&recrs=b&recrs=a&recrs=f&recrs=d&age_v=&gndr=&type=&rslt=0.

[68] Diamantis N., Banerji U. (2016). Antibody-drug conjugates--an emerging class of cancer treatment. Br. J. Cancer.

[69] Bouchard H., Viskov C., Garcia-Echeverria C. (2014). Antibody-drug conjugates-a new wave of cancer drugs. Bioorg. Med. Chem. Lett..

[70] Sun X., Ponte J.F., Yoder N.C., Laleau R., Coccia J., Lanieri L., Qiu Q., Wu R., Hong E., Bogalhas M. (2017). Effects of Drug-Antibody Ratio on Pharmacokinetics, Biodistribution, Efficacy, and Tolerability of Antibody-Maytansinoid Conjugates. Bioconj. Chem..

[71] Weckbecker G., Lewis I., Albert R., Schmid H.A., Hoyer D., Bruns C. (2003). Opportunities in somatostatin research: Biological, chemical and therapeutic aspects. Nat. Rev. Drug Discov..

[72] Keskin O., Yalcin S. (2013). A review of the use of somatostatin analogs in oncology. Onco Targets.

[73] Spada F., Valente M. (2016). Review of recents advances in medical treatment for neuroendocrine neoplasms: Somatostatin analogs and chemotherapy. J. Cancer Metastasis Treat..

[74] Moller L.N., Stidsen C.E., Hartmann B., Holst J.J. (2003). Somatostatin receptors. Biochim. Biophys. Acta.

[75] De Jong M., Valkema R., Jamar F., Kvols L.K., Kwekkeboom D.J., Breeman W.A., Bakker W.H., Smith C., Pauwels S., Krenning E.P. (2002). Somatostatin receptor-targeted radionuclide therapy of tumors: Preclinical and clinical findings. Semin. Nucl. Med..

[76] Pinato D.J., Black J.R., Ramaswami R., Tan T.M., Adjogatse D., Sharma R. (2016). Peptide receptor radionuclide therapy for metastatic paragangliomas. Med. Oncol..

[77] PRRT. http://www.prrtinfo.org/prrt.

[78] Krenning E.P., Kooij P.P., Bakker W.H., Breeman W.A., Postema P.T., Kwekkeboom D.J., Oei H.Y., de Jong M., Visser T.J., Reijs A.E. (1994). Radiotherapy with a radiolabeled somatostatin analogue, [111In-DTPA-DPhe1]- octreotide: A case history. Ann. N. Y. Acad. Sci..

[79] Hörsch D., Ezziddin S., Haug A., Gratz K.F., Dunkelmann S., Miederer M., Schreckenberger M., Krause B.J., Bengel F.M., Bartenstein P. (2016). Effectiveness and side-effects of peptide receptor radionuclide therapy for neuroendocrine neoplasms in Germany: A multi-institutional registry study with prospective follow-up. Eur. J. Cancer.

[80] FDA Approves Lutetium Lu 177 Dotatate for Treatment of GEP-NETS. https://www.fda.gov/drugs/informationondrugs/approveddrugs/ucm594105.htm.

[81] Lutathera. http://www.ema.europa.eu/ema/index.jsp?curl=pages/medicines/human/medicines/004123/human_med_002163.jsp&mid=WC0b01ac058001d1244.

[82] Emmett L., Willowson K., Violet J., Shin J., Blanksby A., Lee J. (2017). Lutetium (177) PSMA radionuclide therapy for men with prostate cancer: A review of the current literature and discussion of practical aspects of therapy. J. Med. Radiat. Sci..

[83] Bodei L., Mueller-Brand J., Baum R.P., Pavel M.E., Horsch D., O’Dorisio M.S., O’Dorisio T.M., Howe J.R., Cremonesi M., Kwekkeboom D.J. (2013). The joint IAEA, EANM, and SNMMI practical guidance on peptide receptor radionuclide therapy (PRRNT) in neuroendocrine tumours. Eur. J. Nucl. Med. Mol. Imaging.

[84] Van Essen M., Krenning E.P., De Jong M., Valkema R., Kwekkeboom D.J. (2007). Peptide Receptor Radionuclide Therapy with radiolabelled somatostatin analogues in patients with receptor positive tumours. Acta Oncol..

[85] Jamous M., Haberkorn U., Mier W. (2013). Synthesis of peptide radiopharmaceuticals for the therapy and diagnosis of tumor diseases. Molecules.

[86] Reubi J.C., Schär J.C., Waser B., Wenger S., Heppeler A., Schmitt J.S., Mäcke H.R. (2000). Affinity profiles for human somatostatin receptor subtypes SST1-SST5 of somatostatin radiotracers selected for scintigraphic and radiotherapeutic use. Eur. J. Nucl. Med..

[87] Kwekkeboom D.J., de Herder W.W., Kam B.L., van Eijck C.H., van Essen M., Kooij P.P., Feelders R.A., van Aken M.O., Krenning E.P. (2008). Treatment with the radiolabeled somatostatin analog [177 Lu-DOTA 0,Tyr3]octreotate: Toxicity, efficacy, and survival. J. Clin. Oncol..

[88] Strosberg J., El-Haddad G., Wolin E., Hendifar A., Yao J., Chasen B., Mittra E., Kunz P.L., Kulke M.H., Jacene H. (2017). Phase 3 Trial of (177)Lu-Dotatate for Midgut Neuroendocrine Tumors. N. Engl. J. Med..

[89] Schneider F., Tomek W., Grundker C. (2006). Gonadotropin-releasing hormone (GnRH) and its natural analogues: A review. Theriogenology.

[90] Millar R.P., Lu Z.L., Pawson A.J., Flanagan C.A., Morgan K., Maudsley S.R. (2004). Gonadotropin-releasing hormone receptors. Endocr. Rev..

[91] Madhunapantula S.V., Mosca P., Robertson G.P. (2010). Steroid hormones drive cancer development. Cancer Biol..

[92] Capper C.P., Rae J.M., Auchus R.J. (2016). The Metabolism, Analysis, and Targeting of Steroid Hormones in Breast and Prostate Cancer. Horm. Cancer.

[93] Perrett R.M., McArdle C.A. (2013). Molecular mechanisms of gonadotropin-releasing hormone signaling integrating cyclic nucleotides into the network. Front. Endocrinol. (Lausanne).

[94] Bolton E.M., Lynch T.H. (2018). Are all gonadotropin-releasing hormone agonists equivalent for the treatment of prostate cancer? A systematic review. BJU Int..

[95] Cheng C.K., Leung P.C. (2005). Molecular biology of gonadotropin-releasing hormone (GnRH)-I, GnRH-II, and their receptors in humans. Endocr. Rev..

[96] Westphalen S., Kotulla G., Kaiser F., Krauss W., Werning G., Elsasser H.P., Nagy A., Schulz K.D., Grundker C., Schally A.V. (2000). Receptor mediated antiproliferative effects of the cytotoxic LHRH agonist AN-152 in human ovarian and endometrial cancer cell lines. Int. J. Oncol..

[97] Letsch M., Schally A.V., Szepeshazi K., Halmos G., Nagy A. (2003). Preclinical evaluation of targeted cytotoxic luteinizing hormone-releasing hormone analogue AN-152 in androgen-sensitive and insensitive prostate cancers. Clin. Cancer Res..

[98] Liu S.V., Tsao-Wei D.D., Xiong S., Groshen S., Dorff T.B., Quinn D.I., Tai Y.C., Engel J., Hawes D., Schally A.V. (2014). Phase I, dose-escalation study of the targeted cytotoxic LHRH analog AEZS-108 in patients with castration- and taxane-resistant prostate cancer. Clin. Cancer Res..

[99] Emons G., Kaufmann M., Gorchev G., Tsekova V., Grundker C., Gunthert A.R., Hanker L.C., Velikova M., Sindermann H., Engel J. (2010). Dose escalation and pharmacokinetic study of AEZS-108 (AN-152), an LHRH agonist linked to doxorubicin, in women with LHRH receptor-positive tumors. Gynecol. Oncol..

[100] Engel J., Emons G., Pinski J., Schally A.V. (2012). AEZS-108: A targeted cytotoxic analog of LHRH for the treatment of cancers positive for LHRH receptors. Expert Opin. Investig. Drugs.

[101] Yu S.S., Athreya K., Liu S.V., Schally A.V., Tsao-Wei D., Groshen S., Quinn D.I., Dorff T.B., Xiong S., Engel J. (2017). A Phase II Trial of AEZS-108 in Castration- and Taxane-Resistant Prostate Cancer. Clin. Genitourin. Cancer.

[102] Emons G., Gorchev G., Harter P., Wimberger P., Stahle A., Hanker L., Hilpert F., Beckmann M.W., Dall P., Grundker C. (2014). Efficacy and safety of AEZS-108 (LHRH agonist linked to doxorubicin) in women with advanced or recurrent endometrial cancer expressing LHRH receptors: A multicenter phase 2 trial (AGO-GYN5). Int. J. Gynecol. Cancer.

[103] Emons G., Gorchev G., Sehouli J., Wimberger P., Stahle A., Hanker L., Hilpert F., Sindermann H., Grundker C., Harter P. (2014). Efficacy and safety of AEZS-108 (INN: Zoptarelin doxorubicin acetate) an LHRH agonist linked to doxorubicin in women with platinum refractory or resistant ovarian cancer expressing LHRH receptors: A multicenter phase II trial of the ago-study group (AGO GYN 5). Gynecol. Oncol..

[104] Zoptarelin Doxorubicin Falls Short in Phase III Endometrial Cancer Trial. https://www.onclive.com/web-exclusives/zoptarelin-doxorubicin-falls-short-in-phase-iii-endometrial-cancer-trial.

[105] Hennenfent K.L., Govindan R. (2006). Novel formulations of taxanes: A review. Old wine in a new bottle?. Ann. Oncol..

[106] Spencer C.M., Faulds D. (1994). Paclitaxel. A review of its pharmacodynamic and pharmacokinetic properties and therapeutic potential in the treatment of cancer. Drugs.

[107] Demeule M., Regina A., Che C., Poirier J., Nguyen T., Gabathuler R., Castaigne J.P., Beliveau R. (2008). Identification and design of peptides as a new drug delivery system for the brain. J. Pharm. Exp..

[108] Shao K., Huang R., Li J., Han L., Ye L., Lou J., Jiang C. (2010). Angiopep-2 modified PE-PEG based polymeric micelles for amphotericin B delivery targeted to the brain. J. Control. Release.

[109] Regina A., Demeule M., Che C., Lavallee I., Poirier J., Gabathuler R., Beliveau R., Castaigne J.P. (2008). Antitumour activity of ANG1005, a conjugate between paclitaxel and the new brain delivery vector Angiopep-2. Br. J. Pharm..

[110] Kurzrock R., Gabrail N., Chandhasin C., Moulder S., Smith C., Brenner A., Sankhala K., Mita A., Elian K., Bouchard D. (2012). Safety, pharmacokinetics, and activity of GRN1005, a novel conjugate of angiopep-2, a peptide facilitating brain penetration, and paclitaxel, in patients with advanced solid tumors. Mol. Cancer Ther..

[111] Drappatz J., Brenner A., Wong E.T., Eichler A., Schiff D., Groves M.D., Mikkelsen T., Rosenfeld S., Sarantopoulos J., Meyers C.A. (2013). Phase I study of GRN1005 in recurrent malignant glioma. Clin. Cancer Res..

[112] Bertrand Y., Currie J.C., Poirier J., Demeule M., Abulrob A., Fatehi D., Stanimirovic D., Sartelet H., Castaigne J.P., Beliveau R. (2011). Influence of glioma tumour microenvironment on the transport of ANG1005 via low-density lipoprotein receptor-related protein 1. Br. J. Cancer.

[113] Li F., Tang S.C. (2017). Targeting metastatic breast cancer with ANG1005, a novel peptide-paclitaxel conjugate that crosses the blood-brain-barrier (BBB). Genes Dis..

[114] https://clinicaltrials.gov/ct2/results?term=ANG1005&age_v=&gndr=&type=&rslt=&phase=1&Search=Apply.

[115] Angiochem’s ANG1005 Received Orphan Drug Designation from FDA for the Treatment of Glioblastoma Multiform. http://angiochem.com/angiochem%E2%80%99s-ang1005-received-orphan-drug-designation-fda-treatment-glioblastoma-multiform.

[116] https://clinicaltrials.gov/ct2/results?cond=&term=NCT03613181&cntry=&state=&city=&dist=.

[117] Quynh Doan N.T., Christensen S.B. (2015). Thapsigargin, Origin, Chemistry, Structure-Activity Relationships and Prodrug Development. Curr. Pharm. Des..

[118] Denmeade S.R., Isaacs J.T. (2012). Engineering enzymatically activated “molecular grenades” for cancer. Oncotarget.

[119] Denmeade S.R., Mhaka A.M., Rosen D.M., Brennen W.N., Dalrymple S., Dach I., Olesen C., Gurel B., Demarzo A.M., Wilding G. (2012). Engineering a prostate-specific membrane antigen-activated tumor endothelial cell prodrug for cancer therapy. Sci. Transl. Med..

[120] Andersen T.B., Lopez C.Q., Manczak T., Martinez K., Simonsen H.T. (2015). Thapsigargin--from Thapsia L. to mipsagargin. Molecules.

[121] Silver D.A., Pellicer I., Fair W.R., Heston W.D., Cordon-Cardo C. (1997). Prostate-specific membrane antigen expression in normal and malignant human tissues. Clin. Cancer Res..

[122] Kinoshita Y., Kuratsukuri K., Landas S., Imaida K., Rovito P.M., Wang C.Y., Haas G.P. (2006). Expression of prostate-specific membrane antigen in normal and malignant human tissues. World J. Surg..

[123] Mahalingam D., Wilding G., Denmeade S., Sarantopoulas J., Cosgrove D., Cetnar J., Azad N., Bruce J., Kurman M., Allgood V.E. (2016). Mipsagargin, a novel thapsigargin-based PSMA-activated prodrug: Results of a first-in-man phase I clinical trial in patients with refractory, advanced or metastatic solid tumours. Br. J. Cancer.

[124] https://clinicaltrials.gov/ct2/results?cond=&term=G202&cntry=&state=&city=&dist=.

[125] Dissanayake S., Denny W.A., Gamage S., Sarojini V. (2017). Recent developments in anticancer drug delivery using cell penetrating and tumor targeting peptides. J. Control. Release.

[126] Kebebe D., Liu Y., Wu Y., Vilakhamxay M., Liu Z., Li J. (2018). Tumor-targeting delivery of herb-based drugs with cell-penetrating/tumor-targeting peptide-modified nanocarriers. Int. J. Nanomed..

[127] Timur S.S., Bhattarai P., Gursoy R.N., Vural I., Khaw B.A. (2017). Design and In Vitro Evaluation of Bispecific Complexes and Drug Conjugates of Anticancer Peptide, LyP-1 in Human Breast Cancer. Pharm. Res..

[128] Engel J.B., Schally A.V., Dietl J., Rieger L., Honig A. (2007). Targeted therapy of breast and gynecological cancers with cytotoxic analogues of peptide hormones. Mol. Pharm..

[129] Schally A.V., Nagy A. (1999). Cancer chemotherapy based on targeting of cytotoxic peptide conjugates to their receptors on tumors. Eur. J. Endocrinol..

[130] Nagy A., Schally A.V., Halmos G., Armatis P., Cai R.Z., Csernus V., Kovacs M., Koppan M., Szepeshazi K., Kahan Z. (1998). Synthesis and biological evaluation of cytotoxic analogs of somatostatin containing doxorubicin or its intensely potent derivative, 2-pyrrolinodoxorubicin. Proc. Natl. Acad. Sci. USA.

[131] Engel J.B., Schally A.V., Halmos G., Baker B., Nagy A., Keller G. (2005). Targeted cytotoxic bombesin analog AN-215 effectively inhibits experimental human breast cancers with a low induction of multi-drug resistance proteins. Endocr. Relat. Cancer.

[132] Nagy A., Armatis P., Cai R.Z., Szepeshazi K., Halmos G., Schally A.V. (1997). Design, synthesis, and in vitro evaluation of cytotoxic analogs of bombesin-like peptides containing doxorubicin or its intensely potent derivative, 2-pyrrolinodoxorubicin. Proc. Natl. Acad. Sci. USA.

[133] Ibanez-Costa A., Lopez-Sanchez L.M., Gahete M.D., Rivero-Cortes E., Vazquez-Borrego M.C., Galvez M.A., de la Riva A., Venegas-Moreno E., Jimenez-Reina L., Moreno-Carazo A. (2017). BIM-23A760 influences key functional endpoints in pituitary adenomas and normal pituitaries: Molecular mechanisms underlying the differential response in adenomas. Sci. Rep..

[134] Florio T., Barbieri F., Spaziante R., Zona G., Hofland L.J., van Koetsveld P.M., Feelders R.A., Stalla G.K., Theodoropoulou M., Culler M.D. (2008). Efficacy of a dopamine-somatostatin chimeric molecule, BIM-23A760, in the control of cell growth from primary cultures of human non-functioning pituitary adenomas: A multi-center study. Endocr. Relat. Cancer.

[135] Jaquet P., Gunz G., Saveanu A., Dufour H., Taylor J., Dong J., Kim S., Moreau J.P., Enjalbert A., Culler M.D. (2005). Efficacy of chimeric molecules directed towards multiple somatostatin and dopamine receptors on inhibition of GH and prolactin secretion from GH-secreting pituitary adenomas classified as partially responsive to somatostatin analog therapy. Eur. J. Endocrinol..

[136] Bakker W.H., Albert R., Bruns C., Breeman W.A., Hofland L.J., Marbach P., Pless J., Pralet D., Stolz B., Koper J.W. (1991). [111In-DTPA-D-Phe1]-octreotide, a potential radiopharmaceutical for imaging of somatostatin receptor-positive tumors: Synthesis, radiolabeling and in vitro validation. Life Sci..

[137] Forssell-Aronsson E., Bernhardt P., Nilsson O., Tisell L.E., Wangberg B., Ahlman H. (2004). Biodistribution data from 100 patients i.v. injected with 111In-DTPA-D-Phe1-octreotide. Acta Oncol..

[138] Northfelt D.W., Allred J.B., Liu H., Hobday T.J., Rodacker M.W., Lyss A.P., Fitch T.R., Perez E.A., North Central Cancer Treatment G. (2014). Phase 2 trial of paclitaxel polyglumex with capecitabine for metastatic breast cancer. Am. J. Clin. Oncol..

[139] Singer J.W. (2005). Paclitaxel poliglumex (XYOTAX, CT-2103): A macromolecular taxane. J. Control. Release.

[140] O’Brien M.E., Socinski M.A., Popovich A.Y., Bondarenko I.N., Tomova A., Bilynsky B.T., Hotko Y.S., Ganul V.L., Kostinsky I.Y., Eisenfeld A.J. (2008). Randomized phase III trial comparing single-agent paclitaxel Poliglumex (CT-2103, PPX) with single-agent gemcitabine or vinorelbine for the treatment of PS 2 patients with chemotherapy-naive advanced non-small cell lung cancer. J. Thorac. Oncol..

[141] Langer C.J., O’Byrne K.J., Socinski M.A., Mikhailov S.M., Lesniewski-Kmak K., Smakal M., Ciuleanu T.E., Orlov S.V., Dediu M., Heigener D. (2008). Phase III trial comparing paclitaxel poliglumex (CT-2103, PPX) in combination with carboplatin versus standard paclitaxel and carboplatin in the treatment of PS 2 patients with chemotherapy-naive advanced non-small cell lung cancer. J. Thorac. Oncol..

[142] Paz-Ares L., Ross H., O’Brien M., Riviere A., Gatzemeier U., Von Pawel J., Kaukel E., Freitag L., Digel W., Bischoff H. (2008). Phase III trial comparing paclitaxel poliglumex vs. docetaxel in the second-line treatment of non-small-cell lung cancer. Br. J. Cancer.

[143] Curtis K.K., Sarantopoulos J., Northfelt D.W., Weiss G.J., Barnhart K.M., Whisnant J.K., Leuschner C., Alila H., Borad M.J., Ramanathan R.K. (2014). Novel LHRH-receptor-targeted cytolytic peptide, EP-100: First-in-human phase I study in patients with advanced LHRH-receptor-expressing solid tumors. Cancer Chemother. Pharm..

[144] Leuschner C., Coulter A., Keener J., Alila H. (2017). Targeted Oncolytic Peptide for Treatment of Ovarian Cancers. Int. J. Cancer Res. Mol. Mech..

[145] https://clinicaltrials.gov/ct2/results?cond=&term=NCT01485848&cntry=&state=&city=&dist=.

[146] Rothbard J.B., Garlington S., Lin Q., Kirschberg T., Kreider E., McGrane P.L., Wender P.A., Khavari P.A. (2000). Conjugation of arginine oligomers to cyclosporin A facilitates topical delivery and inhibition of inflammation. Nat. Med..

[147] Ciclosporin–Cellgate. https://adisinsight.springer.com/drugs/800018283.

[148] KAI Pharmaceuticals Initiates Phase 1 Trial of KAI-1455 for Ischemic Injury. https://www.businesswire.com/news/home/20070504005145/en/KAI-Pharmaceuticals-Initiates-Phase-1-Trial-KAI-1455.

[149] Moodie J.E., Bisley E.J., Huang S., Pickthorn K., Bell G. (2013). A single-center, randomized, double-blind, active, and placebo-controlled study of KAI-1678, a novel PKC-epsilon inhibitor, in the treatment of acute postoperative orthopedic pain. Pain Med..

[150] Cousins M.J., Pickthorn K., Huang S., Critchley L., Bell G. (2013). The safety and efficacy of KAI-1678- an inhibitor of epsilon protein kinase C (epsilonPKC)-versus lidocaine and placebo for the treatment of postherpetic neuralgia: A crossover study design. Pain Med..

[151] https://clinicaltrials.gov/ct2/results?cond=&term=kai-1678&cntry=&state=&city=&dist=.

[152] Miyaji Y., Walter S., Chen L., Kurihara A., Ishizuka T., Saito M., Kawai K., Okazaki O. (2011). Distribution of KAI-9803, a novel delta-protein kinase C inhibitor, after intravenous administration to rats. Drug Metab. Dispos..

[153] Bates E., Bode C., Costa M., Gibson C.M., Granger C., Green C., Grimes K., Harrington R., Huber K., Direct Inhibition of delta-Protein Kinase C Enzyme to Limit Total Infarct Size in Acute Myocardial Infarction (DELTA MI) Investigators (2008). Intracoronary KAI-9803 as an adjunct to primary percutaneous coronary intervention for acute ST-segment elevation myocardial infarction. Circulation.

[154] https://clinicaltrials.gov/ct2/results?cond=&term=KAI-9803&cntry=&state=&city=&dist=.

[155] https://clinicaltrials.gov/ct2/results?cond=&term=XG-102&cntry=&state=&city=&dist=.

[156] http://www.xigenpharma.com/clinical-trials.

[157] Chiquet C., Aptel F., Creuzot-Garcher C., Berrod J.P., Kodjikian L., Massin P., Deloche C., Perino J., Kirwan B.A., de Brouwer S. (2017). Postoperative Ocular Inflammation: A Single Subconjunctival Injection of XG-102 Compared to Dexamethasone Drops in a Randomized Trial. Am. J. Ophthalmol..

[158] Beydoun T., Deloche C., Perino J., Kirwan B.A., Combette J.M., Behar-Cohen F. (2015). Subconjunctival injection of XG-102, a JNK inhibitor peptide, in patients with intraocular inflammation: A safety and tolerability study. J. Ocul. Pharm..

[159] Coriat R., Faivre S.J., Mir O., Dreyer C., Ropert S., Bouattour M., Desjardins R., Goldwasser F., Raymond E. (2016). Pharmacokinetics and safety of DTS-108, a human oligopeptide bound to SN-38 with an esterase-sensitive cross-linker in patients with advanced malignancies: A Phase I study. Int. J. Nanomed..

[160] Meyer-Losic F., Nicolazzi C., Quinonero J., Ribes F., Michel M., Dubois V., de Coupade C., Boukaissi M., Chene A.S., Tranchant I. (2008). DTS-108, a novel peptidic prodrug of SN38: In vivo efficacy and toxicokinetic studies. Clin. Cancer Res..

[161] Schoffski P., Delord J.P., Brain E., Robert J., Dumez H., Gasmi J., Trouet A. (2017). First-in-man phase I study assessing the safety and pharmacokinetics of a 1-h intravenous infusion of the doxorubicin prodrug DTS-201 every 3 weeks in patients with advanced or metastatic solid tumours. Eur. J. Cancer.

[162] Ravel D., Dubois V., Quinonero J., Meyer-Losic F., Delord J., Rochaix P., Nicolazzi C., Ribes F., Mazerolles C., Assouly E. (2008). Preclinical toxicity, toxicokinetics, and antitumoral efficacy studies of DTS-201, a tumor-selective peptidic prodrug of doxorubicin. Clin. Cancer Res..

[163] https://www.clinicaltrialsregister.eu/ctr-search/search?query=DTS-201.

[164] https://clinicaltrials.gov/ct2/show/NCT03486730.

[165] https://clinicaltrials.gov/ct2/show/NCT03511664.

[166] Gupte A.A., Pownall H.J., Hamilton D.J. (2015). Estrogen: An emerging regulator of insulin action and mitochondrial function. J. Diabetes Res..

[167] Mauvais-Jarvis F. (2011). Estrogen and androgen receptors: Regulators of fuel homeostasis and emerging targets for diabetes and obesity. Trends Endocrinol. Metab..

[168] Pereira R.I., Casey B.A., Swibas T.A., Erickson C.B., Wolfe P., Van Pelt R.E. (2015). Timing of Estradiol Treatment After Menopause May Determine Benefit or Harm to Insulin Action. J. Clin. Endocrinol. Metab..

[169] Bonds D.E., Lasser N., Qi L., Brzyski R., Caan B., Heiss G., Limacher M.C., Liu J.H., Mason E., Oberman A. (2006). The effect of conjugated equine oestrogen on diabetes incidence: The Women’s Health Initiative randomised trial. Diabetologia.

[170] Rossouw J.E., Anderson G.L., Prentice R.L., LaCroix A.Z., Kooperberg C., Stefanick M.L., Jackson R.D., Beresford S.A., Howard B.V., Johnson K.C. (2002). Risks and benefits of estrogen plus progestin in healthy postmenopausal women: Principal results From the Women’s Health Initiative randomized controlled trial. JAMA.

[171] Kim J.H., Meyers M.S., Khuder S.S., Abdallah S.L., Muturi H.T., Russo L., Tate C.R., Hevener A.L., Najjar S.M., Leloup C. (2014). Tissue-selective estrogen complexes with bazedoxifene prevent metabolic dysfunction in female mice. Mol. Metab..

[172] Barrera J., Chambliss K.L., Ahmed M., Tanigaki K., Thompson B., McDonald J.G., Mineo C., Shaul P.W. (2014). Bazedoxifene and conjugated estrogen prevent diet-induced obesity, hepatic steatosis, and type 2 diabetes in mice without impacting the reproductive tract. Am. J. Physiol. Endocrinol. Metab..

[173] Hansdottir H. (2008). Raloxifene for older women: A review of the literature. Clin. Interv. Aging.

[174] Barrett-Connor E., Mosca L., Collins P., Geiger M.J., Grady D., Kornitzer M., McNabb M.A., Wenger N.K., Investigators R.U.f.T.H.R.T. (2006). Effects of raloxifene on cardiovascular events and breast cancer in postmenopausal women. N. Engl. J. Med..

[175] Finan B., Yang B., Ottaway N., Stemmer K., Muller T.D., Yi C.X., Habegger K., Schriever S.C., Garcia-Caceres C., Kabra D.G. (2012). Targeted estrogen delivery reverses the metabolic syndrome. Nat. Med..

[176] Bullock B.P., Heller R.S., Habener J.F. (1996). Tissue distribution of messenger ribonucleic acid encoding the rat glucagon-like peptide-1 receptor. Endocrinology.

[177] The Human Protein Atlas. https://www.proteinatlas.org/ENSG00000112164-GLP1R/tissue.

[178] Cao X., Xu P., Oyola M.G., Xia Y., Yan X., Saito K., Zou F., Wang C., Yang Y., Hinton A. (2014). Estrogens stimulate serotonin neurons to inhibit binge-like eating in mice. J. Clin. Investig..

[179] Vogel H., Wolf S., Rabasa C., Rodriguez-Pacheco F., Babaei C.S., Stober F., Goldschmidt J., DiMarchi R.D., Finan B., Tschop M.H. (2016). GLP-1 and estrogen conjugate acts in the supramammillary nucleus to reduce food-reward and body weight. Neuropharmacology.

[180] Tiano J.P., Tate C.R., Yang B.S., DiMarchi R., Mauvais-Jarvis F. (2015). Effect of targeted estrogen delivery using glucagon-like peptide-1 on insulin secretion, insulin sensitivity and glucose homeostasis. Sci. Rep..

[181] Schwenk R.W., Baumeier C., Finan B., Kluth O., Brauer C., Joost H.G., DiMarchi R.D., Tschop M.H., Schurmann A. (2015). GLP-1-oestrogen attenuates hyperphagia and protects from beta cell failure in diabetes-prone New Zealand obese (NZO) mice. Diabetologia.

[182] Quarta C., Clemmensen C., Zhu Z., Yang B., Joseph S.S., Lutter D., Yi C.X., Graf E., Garcia-Caceres C., Legutko B. (2017). Molecular Integration of Incretin and Glucocorticoid Action Reverses Immunometabolic Dysfunction and Obesity. Cell Metab..

[183] Grozinsky-Glasberg S., Fraser A., Nahshoni E., Weizman A., Leibovici L. (2006). Thyroxine-triiodothyronine combination therapy versus thyroxine monotherapy for clinical hypothyroidism: Meta-analysis of randomized controlled trials. J. Clin. Endocrinol. Metab..

[184] Mullur R., Liu Y.Y., Brent G.A. (2014). Thyroid hormone regulation of metabolism. Physiol. Rev..

[185] Sinha R.A., Singh B.K., Yen P.M. (2014). Thyroid hormone regulation of hepatic lipid and carbohydrate metabolism. Trends Endocrinol. Metab..

[186] Ochs N., Auer R., Bauer D.C., Nanchen D., Gussekloo J., Cornuz J., Rodondi N. (2008). Meta-analysis: Subclinical thyroid dysfunction and the risk for coronary heart disease and mortality. Ann. Intern. Med..

[187] Baxter J.D., Webb P. (2009). Thyroid hormone mimetics: Potential applications in atherosclerosis, obesity and type 2 diabetes. Nat. Rev. Drug Discov..

[188] Muller T.D., Finan B., Clemmensen C., DiMarchi R.D., Tschop M.H. (2017). The New Biology and Pharmacology of Glucagon. Physiol. Rev..

[189] Finan B., Clemmensen C., Zhu Z., Stemmer K., Gauthier K., Muller L., De Angelis M., Moreth K., Neff F., Perez-Tilve D. (2016). Chemical Hybridization of Glucagon and Thyroid Hormone Optimizes Therapeutic Impact for Metabolic Disease. Cell.

[190] Cox D., Brennan M., Moran N. (2010). Integrins as therapeutic targets: Lessons and opportunities. Nat. Rev. Drug Discov..

[191] Rathinam R., Alahari S.K. (2010). Important role of integrins in the cancer biology. Cancer Metastasis Rev..

[192] Desgrosellier J.S., Cheresh D.A. (2010). Integrins in cancer: Biological implications and therapeutic opportunities. Nat. Rev. Cancer.

[193] Ley K., Rivera-Nieves J., Sandborn W.J., Shattil S. (2016). Integrin-based therapeutics: Biological basis, clinical use and new drugs. Nat. Rev. Drug Discov..

[194] Cox N., Kintzing J.R., Smith M., Grant G.A., Cochran J.R. (2016). Integrin-Targeting Knottin Peptide-Drug Conjugates Are Potent Inhibitors of Tumor Cell Proliferation. Angew. Chem..

[195] Toschi L., Finocchiaro G., Bartolini S., Gioia V., Cappuzzo F. (2005). Role of gemcitabine in cancer therapy. Future Oncol..

[196] Noble S., Goa K.L. (1997). Gemcitabine. A review of its pharmacology and clinical potential in non-small cell lung cancer and pancreatic cancer. Drugs.

[197] Karampelas T., Skavatsou E., Argyros O., Fokas D., Tamvakopoulos C. (2017). Gemcitabine Based Peptide Conjugate with Improved Metabolic Properties and Dual Mode of Efficacy. Mol. Pharm..

[198] Jain N., Smith S.W., Ghone S., Tomczuk B. (2015). Current ADC Linker Chemistry. Pharm. Res..

[199] Lu J., Jiang F., Lu A., Zhang G. (2016). Linkers Having a Crucial Role in Antibody-Drug Conjugates. Int. J. Mol. Sci..

[200] McCombs J.R., Owen S.C. (2015). Antibody drug conjugates: Design and selection of linker, payload and conjugation chemistry. Aaps J..

[201] Gebleux R., Casi G. (2016). Antibody-drug conjugates: Current status and future perspectives. Pharm. Ther..

[202] Kim E.G., Kim K.M. (2015). Strategies and Advancement in Antibody-Drug Conjugate Optimization for Targeted Cancer Therapeutics. Biomol. Ther. (Seoul).

[203] Chari R.V., Miller M.L., Widdison W.C. (2014). Antibody-drug conjugates: An emerging concept in cancer therapy. Angew. Chem..

[204] Tsuchikama K., An Z. (2018). Antibody-drug conjugates: Recent advances in conjugation and linker chemistries. Protein Cell.

[205] Doronina S.O., Bovee T.D., Meyer D.W., Miyamoto J.B., Anderson M.E., Morris-Tilden C.A., Senter P.D. (2008). Novel peptide linkers for highly potent antibody-auristatin conjugate. Bioconj. Chem..

[206] Staben L.R., Koenig S.G., Lehar S.M., Vandlen R., Zhang D., Chuh J., Yu S.F., Ng C., Guo J., Liu Y. (2016). Targeted drug delivery through the traceless release of tertiary and heteroaryl amines from antibody-drug conjugates. Nat. Chem..

[207] Anami Y., Yamazaki C.M., Xiong W., Gui X., Zhang N., An Z., Tsuchikama K. (2018). Glutamic acid-valine-citrulline linkers ensure stability and efficacy of antibody-drug conjugates in mice. Nat. Commun..

[208] Jeffrey S.C., Andreyka J.B., Bernhardt S.X., Kissler K.M., Kline T., Lenox J.S., Moser R.F., Nguyen M.T., Okeley N.M., Stone I.J. (2006). Development and properties of beta-glucuronide linkers for monoclonal antibody-drug conjugates. Bioconj. Chem..

[209] Burke P.J., Senter P.D., Meyer D.W., Miyamoto J.B., Anderson M., Toki B.E., Manikumar G., Wani M.C., Kroll D.J., Jeffrey S.C. (2009). Design, synthesis, and biological evaluation of antibody-drug conjugates comprised of potent camptothecin analogues. Bioconj. Chem..

[210] Gunnoo S.B., Madder A. (2016). Chemical Protein Modification through Cysteine. ChemBioChem.

[211] Gunnoo S.B., Madder A. (2016). Bioconjugation—Using selective chemistry to enhance the properties of proteins and peptides as therapeutics and carriers. Org. Biomol. Chem..

[212] Su D., Kozak K.R., Sadowsky J., Yu S.F., Fourie-O’Donohue A., Nelson C., Vandlen R., Ohri R., Liu L., Ng C. (2018). Modulating Antibody-Drug Conjugate Payload Metabolism by Conjugation Site and Linker Modification. Bioconj. Chem..

[213] Spicer C.D., Davis B.G. (2014). Selective chemical protein modification. Nat. Commun..

[214] Chudasama V., Maruani A., Caddick S. (2016). Recent advances in the construction of antibody-drug conjugates. Nat. Chem..

[215] Schumacher D., Hackenberger C.P., Leonhardt H., Helma J. (2016). Current Status: Site-Specific Antibody Drug Conjugates. J. Clin. Immunol..

[216] Sochaj A.M., Swiderska K.W., Otlewski J. (2015). Current methods for the synthesis of homogeneous antibody-drug conjugates. Biotechnol. Adv..

[217] Calce E., Leone M., Monfregola L., De Luca S. (2013). Chemical modifications of peptide sequences via S-alkylation reaction. Org. Lett..

[218] Lu Y., Huang F., Wang J., Xia J. (2014). Affinity-guided covalent conjugation reactions based on PDZ-peptide and SH3-peptide interactions. Bioconj. Chem..

[219] Calce E., Leone M., Mercurio F.A., Monfregola L., De Luca S. (2015). Solid-Phase S-Alkylation Promoted by Molecular Sieves. Org. Lett..

[220] Doan N.D., Bourgault S., Letourneau M., Fournier A. (2008). Effectiveness of the suzuki-miyaura cross-coupling reaction for solid-phase peptide modification. J. Comb. Chem..

[221] Afonso A., Feliu L., Planas M. (2011). Solid-phase synthesis of biaryl cyclic peptides by borylation and microwave-assisted intramolecular Suzuki–Miyaura reaction. Tetrahedron.

[222] Silvestri A.P., Cistrone P.A., Dawson P.E. (2017). Adapting the Glaser Reaction for Bioconjugation: Robust Access to Structurally Simple, Rigid Linkers. Angew. Chem..

[223] Pagel M., Meier R., Braun K., Wiessler M., Beck-Sickinger A.G. (2016). On-resin Diels-Alder reaction with inverse electron demand: An efficient ligation method for complex peptides with a varying spacer to optimize cell adhesion. Org. Biomol. Chem..

[224] Bartlett S., Spring D.R. (2016). C-H activation: Complex peptides made simple. Nat. Chem..

[225] Oriana S., Cai Y., Bode J.W., Yamakoshi Y. (2017). Synthesis of tri-functionalized MMP2 FRET probes using a chemo-selective and late-stage modification of unprotected peptides. Org. Biomol. Chem..

[226] Decostaire I.E., Lelievre D., Aucagne V., Delmas A.F. (2014). Solid phase oxime ligations for the iterative synthesis of polypeptide conjugates. Org. Biomol. Chem..

[227] VanBrunt M.P., Shanebeck K., Caldwell Z., Johnson J., Thompson P., Martin T., Dong H., Li G., Xu H., D’Hooge F. (2015). Genetically Encoded Azide Containing Amino Acid in Mammalian Cells Enables Site-Specific Antibody-Drug Conjugates Using Click Cycloaddition Chemistry. Bioconj. Chem..

[228] Presolski S.I., Hong V.P., Finn M.G. (2011). Copper-Catalyzed Azide-Alkyne Click Chemistry for Bioconjugation. Curr. Protoc. Chem. Biol..

[229] El-Faham A., Albericio F. (2011). Peptide coupling reagents, more than a letter soup. Chem. Rev..

[230] Li D., Elbert D.L. (2002). The kinetics of the removal of the N-methyltrityl (Mtt) group during the synthesis of branched peptides. J. Pept. Res..

[231] Koniev O., Wagner A. (2015). Developments and recent advancements in the field of endogenous amino acid selective bond forming reactions for bioconjugation. Chem. Soc. Rev..

[232] Anderson G.W., Callahan F.M., Zimmerman J.E. (1967). Synthesis of N-hydroxysuccinimide esters of acyl peptides by the mixed anhydride method. J. Am. Chem. Soc..

[233] Lapidot Y., Rappoport S., Wolman Y. (1967). Use of esters of N-hydroxysuccinimide in the synthesis of N-acylamino acids. J. Lipid Res..

[234] Nagy A., Schally A.V., Armatis P., Szepeshazi K., Halmos G., Kovacs M., Zarandi M., Groot K., Miyazaki M., Jungwirth A. (1996). Cytotoxic analogs of luteinizing hormone-releasing hormone containing doxorubicin or 2-pyrrolinodoxorubicin, a derivative 500–1000 times more potent. Proc. Natl. Acad. Sci. USA.

[235] White C.J., Yudin A.K. (2011). Contemporary strategies for peptide macrocyclization. Nat. Chem..

[236] Basle E., Joubert N., Pucheault M. (2010). Protein chemical modification on endogenous amino acids. Chem. Biol..

[237] Geng Q., Sun X., Gong T., Zhang Z.R. (2012). Peptide-drug conjugate linked via a disulfide bond for kidney targeted drug delivery. Bioconj. Chem..

[238] Song Q., Chuan X., Chen B., He B., Zhang H., Dai W., Wang X., Zhang Q. (2016). A smart tumor targeting peptide-drug conjugate, pHLIP-SS-DOX: Synthesis and cellular uptake on MCF-7 and MCF-7/Adr cells. Drug Deliv..

[239] Witt D. (2008). Recent developments in disulfide bond formation. Synthesis.

[240] Mandal B., Basu B. (2014). Recent advances in S–S bond formation. Rsc Adv..

[241] Le Guern F., Ouk T.S., Ouk C., Vanderesse R., Champavier Y., Pinault E., Sol V. (2018). Lysine Analogue of Polymyxin B as a Significant Opportunity for Photodynamic Antimicrobial Chemotherapy. ACS Med. Chem. Lett..

[242] Torres O.B., Matyas G.R., Rao M., Peachman K.K., Jalah R., Beck Z., Michael N.L., Rice K.C., Jacobson A.E., Alving C.R. (2017). Heroin-HIV-1 (H2) vaccine: Induction of dual immunologic effects with a heroin hapten-conjugate and an HIV-1 envelope V2 peptide with liposomal lipid A as an adjuvant. Npj Vaccines.

[243] Jalah R., Torres O.B., Mayorov A.V., Li F., Antoline J.F., Jacobson A.E., Rice K.C., Deschamps J.R., Beck Z., Alving C.R. (2015). Efficacy, but not antibody titer or affinity, of a heroin hapten conjugate vaccine correlates with increasing hapten densities on tetanus toxoid, but not on CRM197 carriers. Bioconj. Chem..

[244] Ponte J.F., Sun X., Yoder N.C., Fishkin N., Laleau R., Coccia J., Lanieri L., Bogalhas M., Wang L., Wilhelm S. (2016). Understanding How the Stability of the Thiol-Maleimide Linkage Impacts the Pharmacokinetics of Lysine-Linked Antibody-Maytansinoid Conjugates. Bioconj. Chem..

[245] Dovgan I., Kolodych S., Koniev O., Wagner A. (2016). 2-(Maleimidomethyl)-1,3-Dioxanes (MD): A Serum-Stable Self-hydrolysable Hydrophilic Alternative to Classical Maleimide Conjugation. Sci. Rep..

[246] Fontaine S.D., Reid R., Robinson L., Ashley G.W., Santi D.V. (2015). Long-term stabilization of maleimide-thiol conjugates. Bioconj. Chem..

[247] Lyon R.P., Setter J.R., Bovee T.D., Doronina S.O., Hunter J.H., Anderson M.E., Balasubramanian C.L., Duniho S.M., Leiske C.I., Li F. (2014). Self-hydrolyzing maleimides improve the stability and pharmacological properties of antibody-drug conjugates. Nat. Biotechnol..

[248] Schumacher F.F., Nunes J.P., Maruani A., Chudasama V., Smith M.E., Chester K.A., Baker J.R., Caddick S. (2014). Next generation maleimides enable the controlled assembly of antibody-drug conjugates via native disulfide bond bridging. Org. Biomol. Chem..

[249] Maruani A., Smith M.E., Miranda E., Chester K.A., Chudasama V., Caddick S. (2015). A plug-and-play approach to antibody-based therapeutics via a chemoselective dual click strategy. Nat. Commun..

[250] Behrens C.R., Ha E.H., Chinn L.L., Bowers S., Probst G., Fitch-Bruhns M., Monteon J., Valdiosera A., Bermudez A., Liao-Chan S. (2015). Antibody-Drug Conjugates (ADCs) Derived from Interchain Cysteine Cross-Linking Demonstrate Improved Homogeneity and Other Pharmacological Properties over Conventional Heterogeneous ADCs. Mol. Pharm..

[251] Anderson R.J., Li J., Kedzierski L., Compton B.J., Hayman C.M., Osmond T.L., Tang C.W., Farrand K.J., Koay H.F., Almeida C. (2017). Augmenting Influenza-Specific T Cell Memory Generation with a Natural Killer T Cell-Dependent Glycolipid-Peptide Vaccine. ACS Chem. Biol..

[252] Speir M., Authier-Hall A., Brooks C.R., Farrand K.J., Compton B.J., Anderson R.J., Heiser A., Osmond T.L., Tang C.W., Berzofsky J.A. (2017). Glycolipid-peptide conjugate vaccines enhance CD8(+) T cell responses against human viral proteins. Sci. Rep..

[253] Barile E., Wang S., Das S.K., Noberini R., Dahl R., Stebbins J.L., Pasquale E.B., Fisher P.B., Pellecchia M. (2014). Design, synthesis and bioevaluation of an EphA2 receptor-based targeted delivery system. ChemMedChem.

[254] Wang S., Placzek W.J., Stebbins J.L., Mitra S., Noberini R., Koolpe M., Zhang Z., Dahl R., Pasquale E.B., Pellecchia M. (2012). Novel targeted system to deliver chemotherapeutic drugs to EphA2-expressing cancer cells. J. Med. Chem..

[255] Bonaccorso R.L., Chepurny O.G., Becker-Pauly C., Holz G.G., Doyle R.P. (2015). Enhanced Peptide Stability Against Protease Digestion Induced by Intrinsic Factor Binding of a Vitamin B12 Conjugate of Exendin-4. Mol. Pharm..

